# The role of genetic variation in shaping phenotypic responses to diet in aging *Drosophila melanogaster*

**DOI:** 10.1038/s41437-025-00797-3

**Published:** 2025-09-24

**Authors:** Nikolaj Klausholt Bak, Trudy F. C. Mackay, Fabio Morgante, Kåre Lehmann Nielsen, Jeppe Lund Nielsen, Torsten Nygaard Kristensen, Palle Duun Rohde

**Affiliations:** 1 Department of Chemistry and Bioscience, Aalborg University, Aalborg, Denmark; 2 Center for Human Genetics and Department of Genetics and Biochemistry, Clemson University, Greenwood, SC USA; 3 Department of Health Science and Technology, Aalborg University, Aalborg, Denmark

**Keywords:** Quantitative trait, Genome-wide association studies

## Abstract

Nutrition plays a central role in healthy living, however, extensive variability in individual responses to dietary interventions complicates our understanding of its effects. Here we present a comprehensive study utilizing the *Drosophila* Genetic Reference Panel (DGRP), investigating how genetic variation influences responses to diet and aging. We performed quantitative genetic analyses of the impact of reduced nutrient intake on lifespan, locomotor activity, dry weight, and heat knockdown time (HKDT) measured on the same individual flies. We found a significant decrease in lifespan for flies exposed to a restricted diet compared to those on a control diet. Similarly, a notable reduction in dry weight was observed in 7 and 16-day-old flies on the restricted diet compared to the control diet. In contrast, flies on the restricted diet exhibited higher locomotor activity. Additionally, HKDT was found to be age-dependent. Further, we detected significant genotype-by-diet interaction (GDI), genotype-by-age interaction (GAI) and genotype-by-age-by-diet interaction (GADI) for all traits. Thus, environmental factors play a crucial role in shaping trait variation at different ages and diets, and/or distinct genetic variation influences these traits at different ages and diets. Our genome-wide association study also identified a quantitative trait locus for age-dependent dietary response. The observed GDI and GAI indicate that susceptibility to environmental influences changes as organisms age. These findings could have significant implications for understanding the genetic mechanisms underlying dietary responses and aging in *Drosophila melanogaster*, which may inform future research on dietary recommendations and interventions aimed at promoting healthy aging in humans. The identification of associations between DNA sequence variation and age-dependent dietary responses opens new avenues for research into the genetic mechanisms underlying these interactions.

## Introduction

Lifespan and healthspan are defined as the total time an individual lives and the period of life during which individuals are healthy and free from age-related diseases, respectively (Le Bourg 2020; Seals et al. 2016; Kirkland and Peterson 2009). Extending lifespan without improving healthspan can lead to longer periods of poor health and diminished quality of life (Seals et al. 2016; Tissenbaum 2012). In humans, and model species such as mice (*Mus musculus*), roundworms (*Caenorhabditis elegans*), and fruit flies (*Drosophila melanogaster*), it is well established that lifespan and healthspan can be uncoupled (Bak et al. 2023; Fischer et al. 2016; Kirkland and Peterson 2009; Tissenbaum 2012; Wu et al. 2017). This means that the duration of healthy life can be altered without necessarily changing the overall lifespan, and vice versa. Therefore, living longer does not always mean better health late in life, highlighting the importance of studying both lifespan and healthspan to uncover mechanisms affecting each (Kirkland and Peterson 2009; Wilson et al. 2020).

This topic is of general importance and interest to humans, as the average global lifespan has increased markedly by ca. 21 years from 1960 to 2020 (World Bank Group 2022). However, healthspan has not increased at the same pace (Cambois et al. 2013; Jagger et al. 2008; Garmany et al. 2021). This discrepancy poses significant societal challenges because it reduces the quality of life for the elderly and imposes economic burdens on healthcare systems. However, the interest in aging extends beyond humans. For livestock, improving healthspan can enhance animal welfare. Livestock are raised for economic purposes and are often culled when their production declines. By improving the overall quality of life and health of these production animals, it may be possible to extend their productive years. This approach could reduce costs for farmers and enhance the welfare of the animals (Hoffman and Valencak 2020). In wild populations, understanding aging can help in conservation efforts (Comizzoli and Ottinger 2021; Ottinger et al. 2024), and in evolutionary biology studying aging can provide insights into the natural selection processes that shape lifespan and healthspan and trade-offs between traits (Flatt and Partridge 2018; Kirkwood 2005; Krittika and Yadav 2020).

The considerable variation in age-related traits within and across species raises fundamental questions: Why does the average lifespan differ so much between even closely related species? How do we explain why Greenlandic sharks (*Somniosus microcephalus*) can live 500 years and that closely related species such as the Pacific sleeper (*S. pacificus*) ‘only’ live up to 250 years (Matta et al. 2024)? Why do some *D. melanogaster* lines have an average lifespan of 22 days, while others survive up to 80 days (Ivanov et al. 2015)? Additionally, *D. melanogaster* and *C. elegans* individuals with the same genetic background reared under different environmental conditions can vary in lifespan by more than a factor of five (Huang et al. 2020; Urban et al. 2021). These variations arise due to genetic and environmental factors, as well as their interactions.

To investigate the genetic basis of variation in lifespan and healthspan across age classes and nutritional conditions, we used a subset of the *D. melanogaster* Genetic Reference Panel (DGRP), which comprises over 200 inbred lines with full genome sequences (Huang et al. 2014). Using the DGRP in genetic studies provides many advantages, including the rapid decay of linkage disequilibrium in *D. melanogaster* which allows for precise quantitative trait mapping of DNA sequence variants (Huang et al. 2014). Further, the DGRP system allows testing many individuals with the same genetic background in different environments, enabling an understanding of the interaction between genetic variation and environmental conditions (Rohde et al. 2016; Francis et al. 2021; Mackay and Huang 2018; Patel and Talbert 2021; Unckless et al. 2015).

The DGRP has been instrumental in identifying genes that affect phenotypic trait variation for a large range of different quantitative traits (Mackay and Huang 2018), including aging and response to diets, providing potential targets for anti-aging interventions (Huang et al. 2020; Jin et al. 2022; Wilson et al. 2020). Healthspan studies typically use measures such as locomotor activity, stress responses, and body mass as health indicators (Bamia et al. 2010; Fontana and Hu 2014; Klepsatel et al. 2016a; Lushchak et al. 2012; Mroczek et al. 2021; Ruchitha et al. 2024). Locomotor activity reflects the physical fitness and mobility of the flies (Cobb et al. 2020; Mendez et al. 2016), which declines with increased age or disease, making it a valuable healthspan metric (Jordan et al. 2006; Wilson et al. 2020). Rapid iterative negative geotaxis and *Drosophila* activity monitor (DAM) assays measure locomotor ability and overall activity, respectively, while body size indicates nutritional status and constitutes a fitness component influenced by environmental factors (Anagnostou et al. 2010; Bubliy and Loeschcke 2005; Eickelberg et al. 2022; Krittika and Yadav 2022; Reis 2016). Heat stress resistance is another important metric, as higher resistance often correlates with longer lifespan, better overall health and resilience (Badial et al. 2024; Banse et al. 2024; Belyi et al. 2020; Soo et al. 2023).

In this study, we selected locomotor activity, heat knockdown time (HKDT), and dry weight as key traits due to their interrelated nature and their collective ability to provide an understanding of healthspan. Locomotor activity and dry weight are interrelated because both can be influenced by standard metabolic rate. Higher standard metabolic rates can lead to increased locomotor activity and changes in body weight due to energy expenditure (Fernández et al. 1999; Videlier et al. 2021). Similarly, locomotor activity and HKDT are connected through their association with stress responses; flies with higher stress resistance (higher HKDT) may also maintain higher levels of locomotor activity under stressful conditions (Kjærsgaard et al. 2010; Noer et al. 2024). HKDT and dry weight are interrelated as both can reflect the organism’s resilience and fitness under varying environmental conditions (Klepsatel et al. 2016b; Kristensen et al. 2016). Dry weight can indicate the energy reserves of the flies, where higher energy reserves potentially leads to better performance in both locomotor activity and HKDT, as heavy flies have more resources to cope with physical and thermal stress. As flies age, their locomotor activity typically declines (Jones and Grotewiel 2011), which can be linked to changes in body weight and stress resistance (Bak et al. 2023; Ghimire and Kim 2015). By examining these traits together on the same individual flies, we can gain insights into how genetic and environmental factors collectively influence healthspan and aging.

Here, we investigated the effects of a control diet and a restricted diet on lifespan and healthspan metrics across age in *D. melanogaster*; specifically, locomotor activity, HKDT, and dry weight. Our overarching goal was to better understand how diet can be optimized to promote healthy aging. While reduced nutrient intake is known to influence aging and lifespan, its effects are not uniform across individuals, suggesting a genetic component to diet responsiveness. We were particularly interested in whether the same dietary intervention could have beneficial effects in some genetic backrounds but detrimental effects in others. Therefore, we aimed to determine whether (i) there is genetic variation for all traits across diet environments; (ii) assess genotype-by-diet and genotype-by-age interactions for lifespan, dry weight, locomotor activity and HKDT; (iii) identify potential candidate genes associated with environment-specific or pleiotropic allelic effects within the same trait through genome-wide association studies (GWAS).

## Materials and Methods

### Fly stocks and nutritional environments

We used 98 DGRP lines, obtained from the Bloomington *Drosophila* Stock Center (NIH P40OD018537). The DGRP is a collection of inbred lines derived from a natural population of *D. melanogaster* from Raleigh, North Carolina, USA. Each line has undergone 20 generations of full-sib inbreeding, resulting in extremely low genetic variation within lines and genetic variation reflecting the original population distributed between lines (Mackay et al., 2012); Huang et al. 2014). The complete genomes of all DGRP lines have been sequenced to high coverage (average ~27X), identifying a total of 4,565,215 molecular variants, including single or multiple nucleotide polymorphisms, insertions, deletions, and microsatellites.

Prior to the experiments, flies were maintained in a climate-controlled room at 23 °C and 50% relative humidity with a 12:12 h light/dark cycle. They were housed in vials (25 × 95 mm), with 10 flies per vial, and fed a control diet (dry yeast (60 g ∙ L^−^^1^), sucrose (40 g ∙ L^−1^), oatmeal (30 g ∙ L^−1^), agar (18 g ∙ L^−1^), Nipagen (12 mL∙L^−1^) (Nipagin, Sigma-Aldrich, St. Louis, MO, USA), and acetic acid (1.2 mL∙L^−1^)). The experimental condition consisted of adult flies reared on either the control diet (478 calories∙L^−1^) or a restricted diet (119.5 calories∙L^−1^), under the same temperature, humidity, and 12:12 h light/dark cycle as during maintenance, unless otherwise stated. The restricted diet consisted of a 25% dilution of the nutritional content of the control diet and this was obtained by adding the indigestible compound α-cellulose (Product no. 102550125, Sigma-Aldrich, Buchs SG, Switzerland) (Table 1). The concentrations of agar, Nipagin and acetic acid were the same in the two diet types. This restricted diet has previously been shown to have marked negative consequences for traits related to lifespan and healthspan (Bak et al. 2023).

**Table 1:** Nutritional components of the two different medium types.

Medium types	Yeast	Sucrose	Oat	Agar	Nipagin	Acetic acid	Cellulose
Control	60 g/L	40 g/L	30 g/L	18 g/L	12 mL/L	1.2 mL/L	0 g/L
Restricted (25%)	15 g/L	10 g/L	7.5 g/L	18 g/L	12 mL/L	1.2 mL/L	97.5 g/L

The control diet is set to have 100% nutritional value and the nutritional value of the restrictive diet is set relative to that.

### Experimental design

**Fig. 1:**
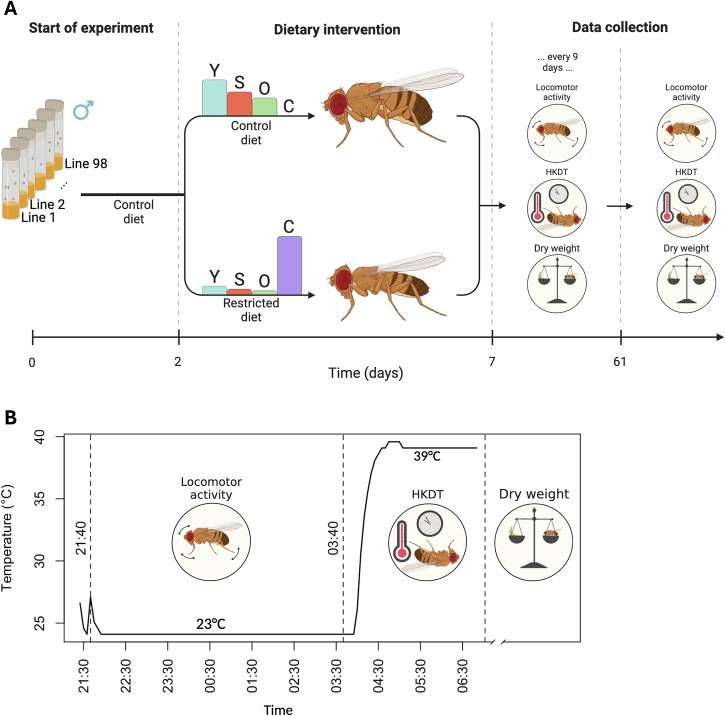
Flowchart of experiment. **A** 98 DGRP lines were raised at 23 °C on a control diet. When the adult flies were 2 days ± 36 h old, they were placed on either a nutritious control diet or a restricted diet. Health metrics, including locomotor activity, heat knock down time (HKDT), and dry weight, were first assessed when the flies were seven days-old and subsequently measured every nine days. From the same pool of flies, mortality was recorded every three days, starting from five day-old flies. Barplots indicate the relative content of the dietary components yeast (Y), sugar (S), oat (O) and cellulose (C). **B** Healthspan metrics were all obtained on the same individual flies. First, locomotor activity was monitored for 6 h at 23 °C. Subsequently, the HKDT assay started with a temperature increase to 39 °C and HKDT was determined as the last recorded activity count using *Drosophila* activity monitors (DAM). Finally, the flies were stored at −80 °C for subsequent measurement of dry weight. The x-axis indicates the time of day.

For each DGRP line, ca. 10 flies were transferred to new vials every third day, for a total of 5-7 transfers. This resulted in 15–20 vials establishing the F1 generation. To avoid introducing early reproduction effects the flies were fed the two different diets during their adult stage, not during development. The F1 generation flies were pooled into five 250 mL bottles per line, each containing 100 flies, to enable consistent density, provided there were enough flies. The flies were transferred to new bottles daily for a total of five days, resulting in up to 25 bottles per line. Upon emergence of the F2 generation, males were retained while females were discarded. Males were used in the experiments because it was not feasible to ensure that the females were virgins, and females have a higher reproduction and age trade-off than males (Carey et al. 2022; Jensen et al. 2015; Strilbytska et al. 2024). Some lines produced over 1000 flies per day, while others yielded only 50–200 flies per day. Therefore, we collected a maximum of 250 flies per day for each line over a 4-day period. On day 5, we pooled all collected flies for each line, representing a mix of ages 2 days ± 36 h, and randomly selected 660 flies per line from this pool. All lines were then simultaneously placed into three bottles (whenever possible) for each diet type, with each bottle containing exactly 90 flies when possible (Fig. 1A). This resulted in a total of 43,750 flies, with 21,832 flies reared on the control diet, and 21,918 reared on the restricted diet. A total of 6,338 flies were used to assess dry weight, 5641 flies to assess locomotor activity, and 3,666 flies to assess HKDT. A total of 4,015 flies were reared on the control diet, and 2,323 flies were reared on the restricted diet. These health metrics were measured on 7 day-old flies and thereafter at 9 day intervals until flies were 61 day-old (*N* = 1561 at test day 7; *N* = 1504 at test day 16; *N* = 1440 at test day 25; *N* = 1126 at test day 34; *N* = 530 at test day 43; *N* = 170 at test day 52 and *N* = 7 at test day 61). Generally, flies had a shorter lifespan when fed on the restricted diet compared to those fed on the control diet, which resulted in an unbalanced data set. The healthspan dataset was balanced by removing lines where all the flies had died on one diet (for the number of flies on each diet and age see Tables S1–S3). Analyses run with the complete data gave similar results, but we had greater power for all health metrics with the balanced dataset (Fig. S1 and Tables S4–S9). With increasing age, fewer individuals were alive for phenotyping, leading to a significant reduction in statistical power. Consequently, data from day 25 and beyond were removed, and we present here the results from analysis of data from days 7 and 16.

### Lifespan assay

For each DGRP line and dietary treatment (see Table 1), we aimed to establish three biological replicates, each consisting of 90 flies housed in individual bottles. While this target was met for most lines, it was not feasible for all. Flies were transferred to fresh bottles containing 60 mL of food medium every three days. During each transfer, mortality was recorded, and deceased individuals were removed from the experiment. This process continued until all flies in all replicates had died.

### Locomotor activity assay

For the first locomotor activity assay, eight seven day-old flies collected across all three replicate bottles per line and nutritional condition were transferred to 5 mm polycarbon tubes (TriKinetics Inc, Waltham, MA USA) containing a pipe cleaner moistened in water in both ends. For subsequent tie-points we aimed to test 16 flies per line and nutritional condition for all other locomotor activity assays. However, flies from some lines survived longer than others and therefore fewer individuals were used at the later activity measurements for short-lived lines (Tables S1–S3). The polycarbon tubes containing flies were added to *Drosophila* Activity Monitors (DAM) (DAM2 Activity Monitor, Trikinetics Inc, Waltham, MA US). The flies were allowed to acclimate in the monitors for 6 h before being placed in a climate-controlled chamber (Binder KB 400, Binder, Tuttlingen, Germany) at 23 °C, with constant light maintained throughout the assay. Locomotor activity was quantified as the number of times individual flies generate an activity count every 10 s for 6 h (9:40 PM to 3:40 AM) (Fig. 1B). Temperature and humidity conditions were recorded every 5 min.

The recording window (9:40 PM–3:40 AM) aligns with the typical rest phase of *D. melanogaster* under a standard light/dark cycle. However, our assay conditions involved continuous light exposure, such that the flies were not experiencing subjective night during the test period. Cho et al. (2016) showed that flies can exhibit meaningful circadian locomotor rhythms without traditional dark phases if the lighting condition includes variation in circadian illuminance. In our study, the constant light environment disrupted normal sleep patterns and enabled measurable locomotor activity, consistent with findings that constant light can induce arrhythmic or altered behaviors in *D. melanogaster*. Moreover, Cho et al. (2016) noted that locomotor rhythms can persist and re-entrain under specific constant-light conditions, especially when considering the spectral quality of the light. This supports that activity measurements obtained during traditionally “inactive” periods can still be biologically meaningful if light conditions are appropriately manipulated.

### Heat stress tolerance

Following the assessment of locomotor activity at 23 °C, the flies were kept in the activity monitors and exposed to 39 °C within the same climate-controlled chamber as where locomotor activity at 23 °C was assessed (Binder KB 400, Binder, Tuttlingen, Germany). Locomotor activity was recorded every 10 s for 2 h (3:40 AM to 5:40 AM), after which all flies were dead (Fig. 1B). The time to death due to heat stress was determined as the last recorded activity count. Temperature and humidity conditions were monitored every 5 min. This assay provided data on HKDT. Individual flies used for locomotor activity and heat tolerance assessments were stored in Eppendorf tubes at −80 °C for subsequent dry weight measurement.

### Dry weight

After assessing heat stress tolerance, the flies were dried for 48 h at 60 °C and subsequently weighed to the nearest 10 μg (Sartorius Quintix35-1S, Satorius, Göttingen, Germany).

### Quantitative genetic analyses

The contributions of age, diet and DGRP line to variation in lifespan, locomotor activity, HKDT and dry weight in the DGRP were assessed separately using mixed model analyses of variance (ANOVA) with a type 3 estimation method utilizing PROC MIXED in SAS (Version 5.2, SAS Institute, Cary, NC). The full mixed model is shown in Eq. 1:

1 $${y}_{{ijk}}=\mu +{L}_{i}+{A}_{j}+{D}_{k}+{L}_{i}\times {A}_{j}+{L}_{i}\times {D}_{k}+{A}_{j}\times {D}_{k}+{L}_{i}\times {A}_{j}\times {D}_{k}+{Rep}\left({L}_{i}\times {A}_{j}\times {D}_{k}\right)+{e}_{{ijk}},$$

where *y*_*ijk*_ is the phenotype; *μ* is the fixed effect of the overall mean; *L*_*i*_ (*i* = 1, 2, …, 98) is the random effect of DGRP line; ${A}_{j}$ (*j* = 1, 2, 3) and *D*_*k*_ (*k* = 1, 2) are the fixed effects of age and diet respectively; *L*_*i*_×*A*_*j*_, *L*_*i*_×*D*_*k*_, *A*_*j*_×*D*_*k*_ and *L*_*i*_×*A*_*j*_×*D*_*k*_ are the interaction effects; *Rep* is the random effect of replicate; and *e*_*ijk*_ is the random residual effect. Broad-sense heritability, ${H}^{2}$, for the full model was estimated as:

2 $${H}^{2}=\frac{{\sigma }_{G}^{2}}{{\sigma }_{G}^{2}+{\sigma }_{e}^{2}}=\frac{{\sigma }_{L}^{2}+{\sigma }_{{LA}}^{2}+{\sigma }_{{LD}}^{2}+{\sigma }_{{LAD}}^{2}}{{\sigma }_{L}^{2}+{\sigma }_{{LA}}^{2}+{\sigma }_{{LD}}^{2}+{\sigma }_{{LAD}}^{2}+{\sigma }_{e}^{2}},$$

where ${\sigma }_{G}^{2}$, ${\sigma }_{L}^{2}$, ${\sigma }_{{LA}}^{2}$, ${\sigma }_{{LD}}^{2}$, ${\sigma }_{{LAD}}^{2}$ and ${\sigma }_{e}^{2}$ are the genetic, among-line, line × age, line × diet, line × age × diet and residual (environmental) variance components, respectively. All combinations of reduced ANOVA models were performed; all ages on separate diets, separate ages on all diets, and separate ages on all separate diets. Standard errors (SE) and confidence intervals (CI) of the ${H}^{2}$ were calculated as in Rohde et al. (2020):

3 $${SE}\left({H}^{2}\right)=\sqrt{{Var}\left({H}^{2}\right)},$$

$${Var}\left({H}^{2}\right)={\left(\frac{\delta {h}^{2}}{\delta {\sigma }_{G}^{2}}\right)}^{2}{\sigma }_{{\sigma }_{G}^{2}}^{2}+{\left(\frac{\delta {h}^{2}}{\delta {\sigma }_{e}^{2}}\right)}^{2}{\sigma }_{{\sigma }_{e}^{2}}^{2}+2\left(\frac{\delta {h}^{2}}{\delta {\sigma }_{G}^{2}}\right)\left(\frac{\delta {h}^{2}}{\delta {\sigma }_{e}^{2}}\right){\sigma }_{{\sigma }_{G,e}}^{2},$$

4 $${CI}={H}^{2}\pm 1.96\cdot {\rm{SE}},$$

where ${\sigma }_{G}^{2}$, ${\sigma }_{e}^{2}$, and ${\sigma }_{G,e}$ are the elements from the asymptotic covariance matrix. Moreover, the partial derivatives are $\frac{\delta {h}^{2}}{\delta {\sigma }_{G}^{2}}=\frac{{\sigma }_{e}^{2}}{{({\sigma }_{G}^{2}+{\sigma }_{G}^{2})}^{2}}$, and $\frac{\delta {h}^{2}}{\delta {\sigma }_{e}^{2}}=\frac{{-\sigma }_{G}^{2}}{{({\sigma }_{G}^{2}+{\sigma }_{G}^{2})}^{2}}$. Genetic correlations, ${\rho }_{{G}_{{ij}}}$, between ages and diets were estimated as Mackay, Falconer (1996):

5 $${\rho }_{{G}_{{ij}}}=\frac{{co}{v}_{{p}_{{ij}}}}{{\sigma }_{{L}_{i}}{\sigma }_{{L}_{j}}},$$

where ${co}{v}_{{p}_{{ij}}}$ is the phenotypic covariance between a trait (*i*) at one diet and age (7 or 16 days) and trait (*j*) at the same or another diet or age. The percent contribution of changes in rank order (% rank), was calculated as in Cockerham (1963):

6 $$\% {rank}=\frac{{\sigma }_{{L}_{i}}{\sigma }_{{L}_{j}}\left(1-{\rho }_{{G}_{{ij}}}\right)}{{\sigma }_{{L}_{i}}{\sigma }_{{L}_{j}}\left(1-{r}_{{G}_{{ij}}}\right)+\frac{{\left({\sigma }_{{L}_{i}}-{\sigma }_{{L}_{j}}\right)}^{2}}{2}},$$

where $\left({\sigma }_{{L}_{i}}{\sigma }_{{L}_{j}}\left(1-{\rho }_{{G}_{{ij}}}\right)\right)$ represents the component of the genotype-by-diet interaction (GDI) or genotype-by-age interaction (GAI) caused by changes in rank order of DGRP line means between the control and restricted diets or between ages 7 and 16 days. $\left({\left({\sigma }_{{L}_{i}}-{\sigma }_{{L}_{j}}\right)}^{2}/2\right)$ is the component of GDI or GAI attributed to differences in the magnitude of between-line genetic variance between the control and restricted diets or ages 7 and 16 days.

Phenotypic correlations were estimated using Spearman correlation coefficients ($\rho$) (Eq. 7):

7 $${\rho }_{{P}_{{ij}}}=\frac{{co}{v}_{{P}_{{ij}}}^{\prime} }{{\sigma }_{i}^{\prime} {\sigma }_{j}^{\prime} },$$

where ${co}{v}_{{Pij}}^{\prime}$ is the covariance of the ranked trait (*i*) at one diet and age (7 or 16 days) and trait (*j*) at the same or another diet or age, while $\sigma {\prime}$ is the ranked standard deviation. Standard errors of the phenotypic and genetic correlations were estimated as in Gnambs (2023):

8 $${SE}=\frac{1-{\rho }^{2}}{\sqrt{n-3}}.$$

Confidence intervals of the genetic correlations were also estimated as in Gnambs (2023):

9 $${CI}=\rho \pm 1.96\cdot {\rm{SE}},$$

while confidence intervals for the phenotypic correlations were calculated using Fisher’s–z transformation (Looney and Hagan 2011):

10 $$z=\frac{1}{2}\cdot \mathrm{ln}\left(\frac{1+\rho }{1-\rho }\right),$$

11 $${z}_{95 \% }=z\pm 1.96\cdot {SE},$$

12 $${CI}=\frac{{e}^{2z}-1}{{e}^{2z}+1},$$

where z is the Fisher’s z-transformed correlation coefficient.

### Survival analysis

The contribution of diet to the age of the DGRP lines was also assessed using a Cox Proportional Hazards model. The risk of events over time, $\lambda \left(t\right)$, was estimated by:

13 $$\lambda \left(t\right)={\lambda }_{0}\left(t\right){e}^{{\beta }_{i}{D}_{i}},$$

where ${\lambda }_{0}\left(t\right)$ is the baseline hazard, ${D}_{i}$ is diet with *i* representing control or restricted diet, and ${\beta }_{i}$ is the regression coefficient. A total of 43,750 flies were used for the survival analysis. We recorded the lifespan of 35,067 flies and censored 8683 flies. Among the censored flies, 303 escaped, 1706 were stuck in the media, 336 were trapped between the flask and stopper, and 6338 were used for healthspan assays.

### Genome-wide association study

Single marker regression analyses were conducted on DGRP line means for lifespan, locomotor activity, HKDT and dry weight separately. We performed a genome-wide association study (GWAS) separately for diets and ages using a mixed model accounting for *Wolbachia* infection status, major chromosomal inversion status (*In(2* *L)t*, *In(2* *R)NS*, *In(3* *R)P*, *In(3* *R)K* and *In(3* *R)Mo*), and polygenic relatedness as described previously (Huang et al. 2020, 2014; Mackay et al., 2012). These analyses assessed the strength of association for additive effects of 1,928,067 polymorphic genetic variants across 98 lines with a minor allele frequency (MAF) greater than 0.05. These analyses were performed for traits with significant genetic components (*p* < 0.05) in the ANOVA models.

We conducted GWAS on DGRP line means within each age group, considering two pairs of environments (control diet *vs*. restricted diet) separately for 7 and 16 day-old flies. Additionally, genotype-by-diet and genotype-by-age interaction effects were investigated using the difference in line means between the conditions as the analysis variable.

The genotype-by-diet interaction for a given trait was defined as the variance in the difference in mean trait values between diets across all lines. Therefore, performing GWAS on these differences for each genetic variant is equivalent to testing for variant-by-diet interaction effects, as described in Huang et al. (2020). Using DGRP line means as the response variable increases the power of estimating broad-sense heritability by reducing environmental variance, as each genotype was measured in a highly replicated experimental setup. This approach is particularly advantageous for traits with low individual-level heritability, as it ensures that heritability is significantly greater than zero, a critical requirement for detecting genetic associations in GWAS (Mackay and Huang 2018).

## Results

We assessed how genetic variation impacts responses to diet and aging using the DGRP. We performed quantitative genetic analyses to evaluate the effects of reduced nutrient intake on lifespan, locomotor activity, dry weight, and heat knockdown time. These measurements were all obtained from the same individual flies.

### Differential effects of diets on lifespan and healthspan

**Fig. 2:**
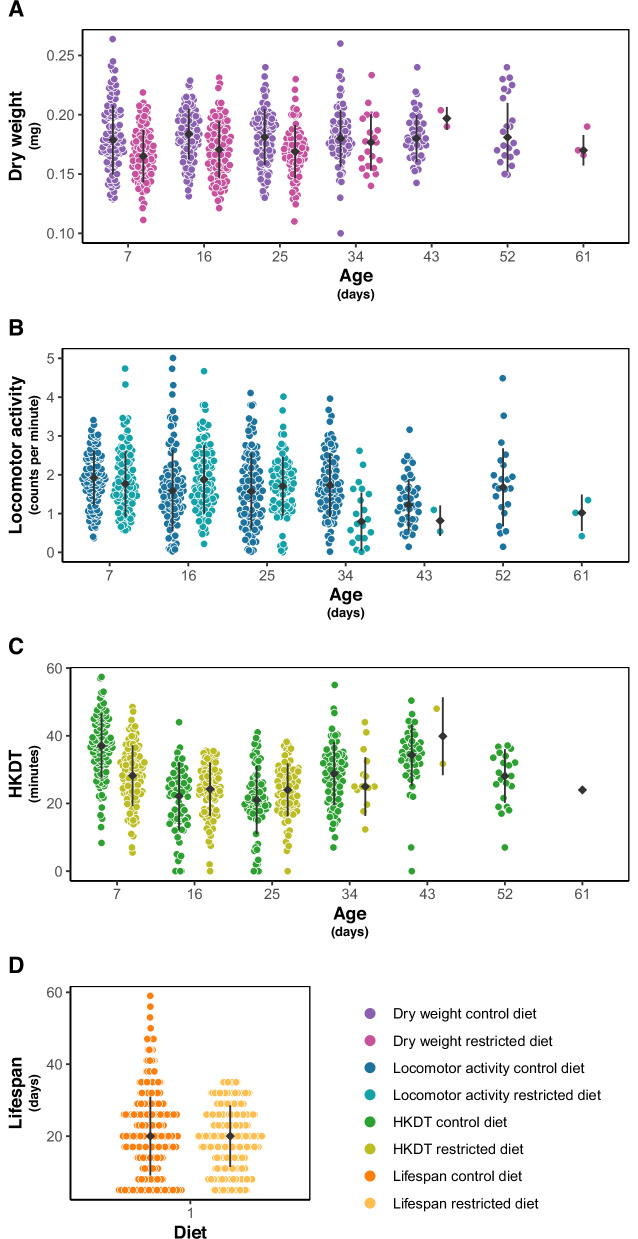
Distribution of line means. Mean (**A**) dry weight (purple), (**B**) locomotor activity (blue) and (**C**) heat knock down time (HKDT) (green) of DGRP lines across seven ages, fed on control diet (dark shades) and restricted diet (light shades), and (**D**) lifespan (orange) also fed on control diet (dark shade) and restricted diet (light shade).

**Fig. 3:**
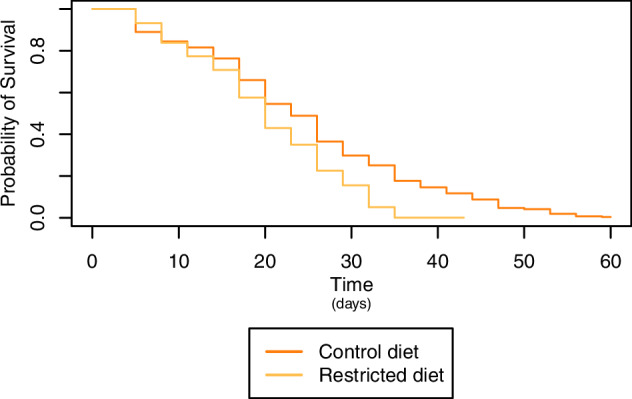
Survival analysis of lifespan. Mean across lines fed control diet (dark orange) and restricted diet (light orange).

Dry weight ranged from 0.07 mg to 0.36 mg, with a mean of 0.18 mg (Figs. 2A and S2A, B, and Tables 2 and S10). A significant difference was observed between the control diet and restricted diet for 7 and 16 day-old flies (*p* < 0.0001; Table S7), with flies on the control diet having, on average, a 7% (0.01 mg) higher dry weight than flies on the restricted diet, while no significant difference was found for the age of the flies across diets (*p* = 0.2052; Table S7). Dry weight varied between lines for combinations of individual diets and ages (Table S7). There was no significant change in dry weight as the flies aged from 7 to 16 days on control diet (*p* = 0.4801, Table S7) or on restricted diet (*p* = 0.2610, Table S7), but there was an interaction effect between line and age on these diets (control diet; *p* < 0.0001, restricted diet; *p* < 0.0001, Table S7). Similar to dry weight, there was a significant decrease in lifespan when flies were exposed to a restricted diet compared to a control diet (Figs. 2D, 3, S2G, H and S3, and Tables 2, 3, S11, and S12). In contrast, locomotor activity was higher for flies exposed to the restricted diet (Figs. 2B and S2C, D, and Tables 2, S8 and S13), while HKDT is age-dependent (Figs. 2C and S3E, F, and Tables 2, S9 and S14). Locomotor activity plasticity with diet is age-dependent, with a significant difference between the two diets observed only for 16 day-old flies. Furthermore, locomotor activity differs across age in a diet-dependent manner, where only flies kept at the control diet showed plasticity with regards to age. HKDT showed plasticity for both diet and age. Similarly, lifespan showed diet-dependent plasticity.

**Table 2:** Average trait means within diet and age groups at the two different diets.

Trait	Control diet		Restricted diet	
	7 day-old	16 day-old	7 day-old	16 day-old
Dry weight	0.182	0.183	0.168	0.171
Locomotor activity	1.890	1.869	1.918	2.022
HKDT	37.11	22.38	29.91	25.40
Lifespan	20.58		19.34

**Table 3:** Summary of the Cox Proportional Hazards model assessing lifespan of DGRP flies fed control and restricted diets.

*Source*	*β*	*se(β)*	*z*	*p-value*
*Diet*	0.25859	0.01117	23.14	<0.0001

### Phenotypic and genetic correlations, and heritability across diets and ages

**Fig. 4:**
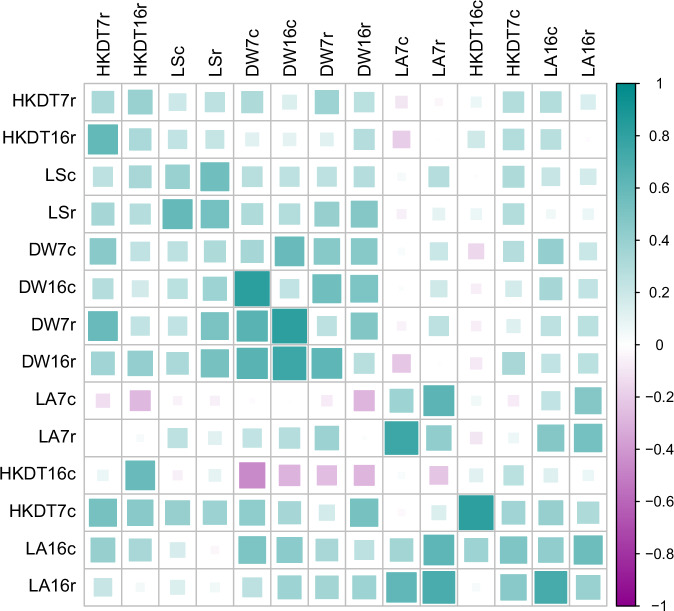
Phenotypic (above diagonal) and genetic (below diagonal) correlations with heritability estimates in the diagonal for all traits. This figure illustrates the linear relationships between traits, age groups, and diet types. The traits are dry weight (DW), lifespan (LS), heat knockdown time (HKDT), and locomotor activity (LA). Age groups are 7 day-old (7) and 16 day-old (16) flies, and diet types are control diet (c) and restricted diet (r). The upper diagonal matrix displays the phenotypic Spearman correlation coefficients between traits, the diagonal contains the estimated heritabilities and the lower diagonal matrix shows the genetic correlations between traits. Color and size indicate the strength of the correlations and the heritability (cyan for positive correlations and magenta for negative correlations, with a larger size indicating a stronger correlation).

To understand the relationships between the various traits, age groups, and diets, we estimated the phenotypic correlations among them. Strong positive correlations between the same traits across different age groups and diet types were observed for all traits (Fig. 4 and Tables S15 and S16). Most of the phenotypic correlations between traits were positive, with the exception of locomotor activity in 7 day-old flies fed the control diet and HKDT in 16 day-old flies fed the control diet, which showed negative correlations with other traits (Fig. 4 and Tables S15 and S16).

**Fig. 5:**
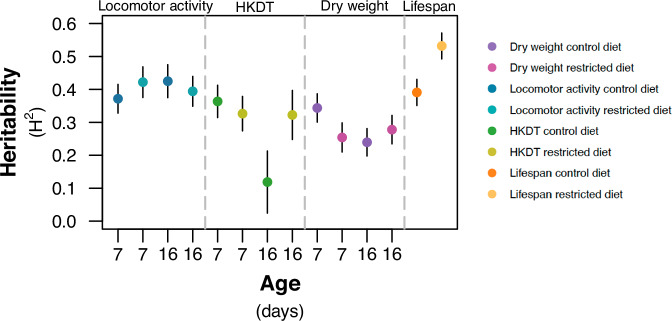
Heritability estimates. This figure illustrates the heritability estimates of locomotor activity (blue), heat knock down time (HKDT) (green) and dry weight (purple) for 7 days old and 16 days old flies, fed on control diet (dark shades) and restricted diet (light shades), and (D) lifespan (orange) flies also fed on control diet (dark shades) and restricted diet (light shades). Points represent the heritability estimate and error bars are standard errors of the mean.

The estimates of broad sense heritability ($\hat{{H}^{2}}$) for dry weight ranged from 0.24 to 0.34, for locomotor activity from 0.37 to 0.43, for HKDT from 0.12 to 0.36 and for lifespan from 0.39 to 0.53 (Figs. 4 and 5, and Tables S15 and S16). Most of the estimated environmental variance components, genetic variance components and heritabilities were significantly different from zero, but not significantly different between diets and ages within traits (Figs. 4 and 5, and Tables S15 and S16).

**Fig. 6:**
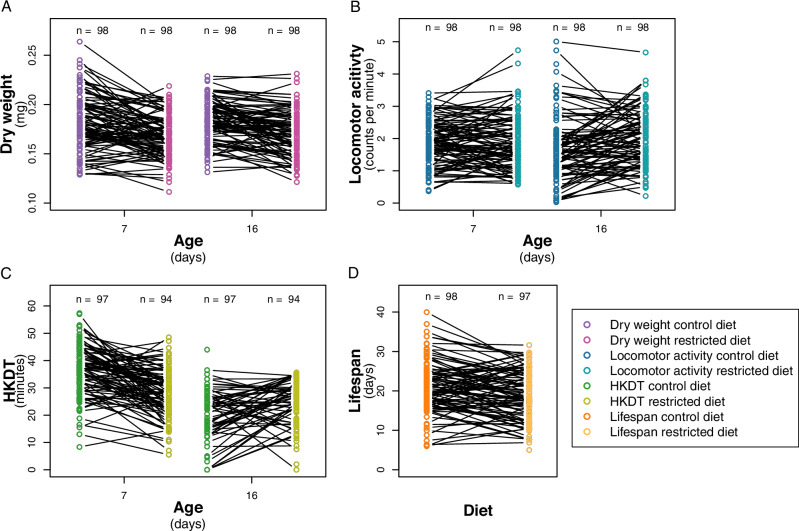
Reaction norm plot. Line means of (**A**) dry weight (purple), (**B**) locomotor activity (blue) and (**C**) heat knock down time (HKDT) (green), fed on control diet (dark shades) and restricted diet (light shades), and (**D**) lifespan (orange) also fed on control diet (dark shades) and restricted diet (light shades). Points represent the mean trait of each DGRP line, and lines connecting points across diets represent the same DGRP line. Numbers (n) of DGRP lines assessed at each age and diet is indicated above the points. Three biological replicates of ~240 flies/replicate were used for all healthspan traits. Sample sizes were 6388, 5641, 3666 and 35,067 flies for dry weight, locomotor activity, HKDT and lifespan, respectively.

The cross-diet genetic correlation for dry weight between control and restricted diets (${\rho }_{{G}_{{Diet}}}$) was 0.645 at 7 days and 0.755 at 16 days (Fig. 4 and Tables S15 and S16). The interaction effect between diet and line was significant at both day 7 (*p* < 0.0001, Table S7) and day 16 (*p* = 0.0030, Table S7). Therefore, the genetic correlations at both ages were signficantly less than unity, indicating genotype-by-diet interaction (GDI) for this trait, i.e., the difference in dry weight between the control and restricted diets significantly varied among the DGRP lines. The significant GDI indicates that the genetic effects on dry weight differ between the two diets, implying that it is not the same genetic makeup that impacts the trait in the two environments. We assessed the extent to which GDI for dry weight was due to changes in between line variance vs. changes in rank order among the DGRP lines and found that the GDI variance was primarily due to changes in rank order of dry weight line means (78.6% at day 7 to 95.8% at day 16) (Figs. 6 and S4, and Table S17).

The cross-age genetic correlation for dry weight of 7 and 16 day-old flies (${\rho }_{{G}_{{Age}}}$) was higher on the control diet (0.823) than the restricted diet (0.623) (Fig. 4 and Tables S15 and S16). Since the interaction effect between age and line was significant both on the control diet (*p* < 0.0001, Table S7) and on the restricted diet (*p* = 0.0003, Table S7), the correlations on both diets were significantly less than unity, indicating genotype-by-age interaction (GAI) for this trait (Fig. S5A). The GAI variance was mostly due to differences in the rank order of line means of dry weight between day 7 and day 16 on the control diet (93.2% rank, Fig. S4 and Table S17) and on the restricted diet (99.2% rank, Fig. S4 and Table S17).

Locomotor activity, HKDT, and lifespan exhibited highly significant interaction effects between both diet and line and age and line, indicating GDI and GAI for these traits as well (Figs. S4 and S5B–D, and Tables S8, S9 and S11). The GDI was primarily influenced by the rank order of line means for locomotor activity, HKDT and lifespan, which ranged from 88.2% to 99.9% (Fig. S4 and Table S17). GAI was also largely due to the rank order of line means for locomotor activity and HKDT with values increasing from 72.1% to 100% (Fig. S4 and Table S17).

### GWAS identifies genetic variants driving diet-dependent effects on dry weight and lifespan

**Fig. 7:**
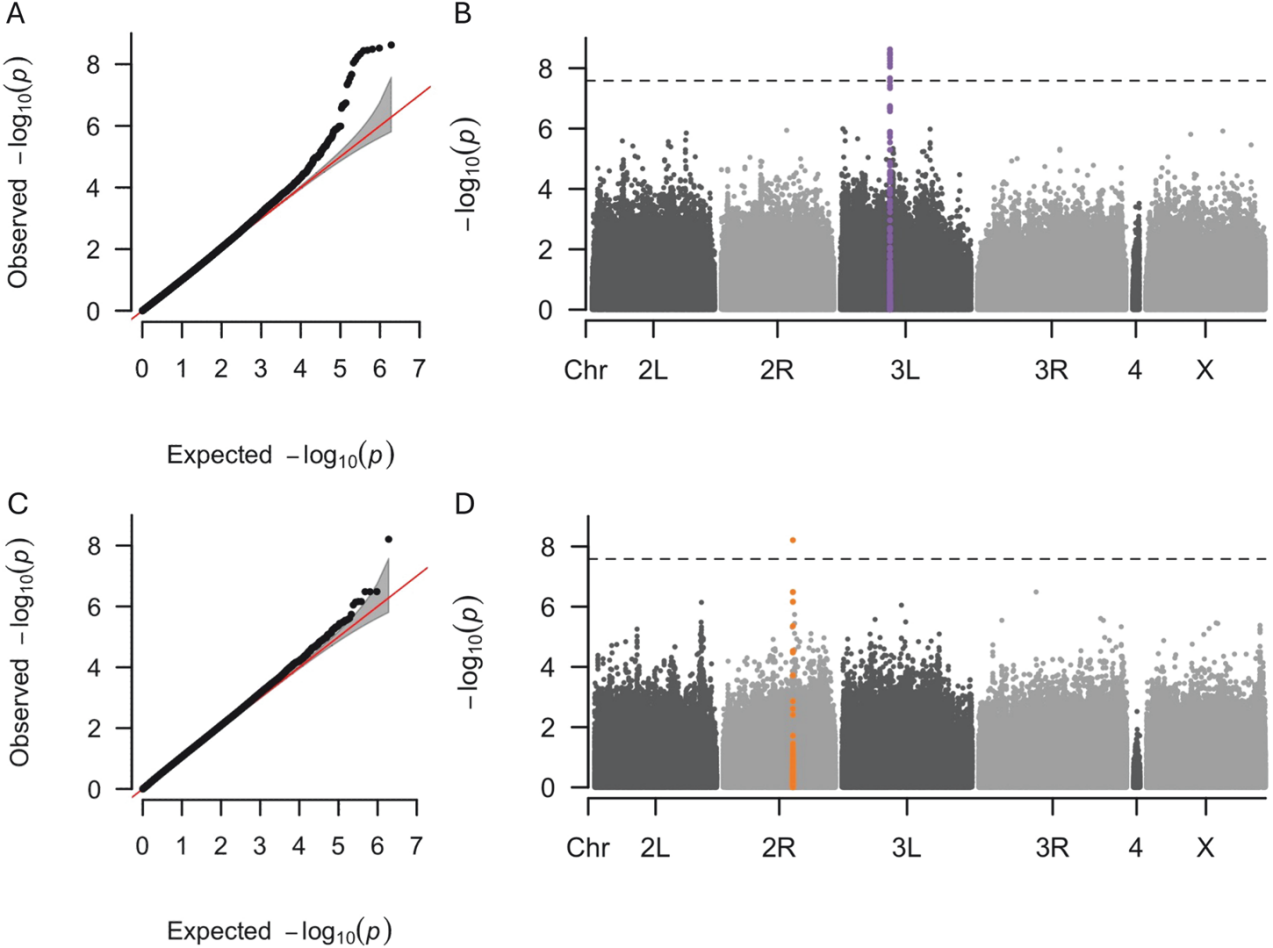
Q-Q and Manhattan plots for GWAS of GDI for dry weight and lifespan. Results of GWAS for the genotype-by-diet interaction for dry weight of 7 day-old flies (**A,** **B**) and genotype-by-diet interaction for lifespan (**C,** **D**). Panels (**A,** **C**) are Q-Q plots comparing the observed -log_10_(*p*) values of each variant to the expected values, with the red line representing the null expectation and the grey area indicating the confidence interval. Panels (**B,** **D**) are Manhattan plots where each point represents a variant. The *y*-axes show the strength of the association between individual variants and GDI for dry weight and lifespan, expressed as -log_10_(*p*). The dashed horizontal lines indicate the significance threshold adjusted for multiple testing using the Bonferroni correction. Variants highlighted for dry weight (purple) and lifespan (orange) indicate the GWAS index variant and all other variants within ± 2500 base pairs of the index variant.

Genotype-by-diet interaction for dry weight showed a strong signal for the variants in the gene encoding Peptidoglycan Recognition Protein LC (*PGRP-LC*) (Fig. 7 and Table S18). *PGRP-LC* is essential for the innate immune response in *D. melanogaster* (Aggarwal and Silverman 2008; Borge-Renberg 2008). No other genetic variants were associated with dry weight, locomotor activity and HKDT (Figs. S6–S8) at the genome-wide significance threshold. However, a single genetic variant within the gene muscleblind (*mbl*) was genome-wide significant for the lifespan genotype-by-diet interaction (Figs. 7C, D and S9 and Table S19). *mbl* encodes an RNA-binding protein that plays a crucial role in RNA metabolism, including alternative splicing, transcript localization, and the biogenesis of miRNA and circRNA (Fernandez-Costa et al. 2011; Li and Millard 2019; Oddo et al. 2016).

## Discussion

Dietary restriction (DR) has been shown to delay aging in numerous species (Le Couteur et al. 2024). While some studies have shown that reduced nutrient intake extends longevity in flies (Durham et al. 2014; Li et al. 2024; Wilson et al. 2020), Bak et al. (2023) found that decreasing nutrient intake reduces both longevity and healthspan. In addition, dietary responses depend on genetic background (Francis et al. 2021; Huang et al. 2020; Patel and Talbert 2021). Diet plays a pivotal role in promoting healthy living, yet individual responses to dietary interventions can vary widely, complicating our understanding of its effects. The aim of the current study was to explore how individual genotypes influence responses to diet and the aging process in *D. melanogaster*. Specifically, we sought to determine whether the same dietary intervention could have beneficial effects in some genetic backgrounds but detrimental effects in others. Using 98 DGRP lines, we examined how a control diet and a restricted diet impacted both lifespan and multiple healthspan metrics, locomotor activity, heat knockdown time (HKDT), and dry weight, measured on the same individuals. This design allowed us to assess genotype-by-diet and genotype-by-age interactions at high resolution and to investigate the genetic architecture underlying variation in both lifespan and healthspan traits. By integrating these data, our study provides novel insights into the genetic basis of diet-dependent aging and highlights the potential for personalized dietary strategies to promote healthy aging.

We found that feeding DGRP flies the restricted diet resulted in an average decrease in mean lifespan compared to flies fed a nutritionally rich control diet (Figs. 2D, 3 and S3, and Tables 2, 3 and S11). This suggests that the restricted diet may have caused malnutrition rather than DR. Nakagawa et al. (2012) conducted a meta-analysis, including a range of species, which showed a U-shaped relationship between the risk of death and nutrient intake, indicating that an intermediate level of calorie intake increases lifespan. Our findings align with the broader understanding that reduced nutrient intake can lead to reduced longevity in some contexts, possibly due to the stress associated with insufficient nutrient intake. Furthermore, mice studies on fasting, defined as a period without calorie consumption, have shown that the positive effects of DR can, in some cases, be attributed more to the effects of fasting itself rather than just the reduction in calorie intake (Pak et al. 2021; Solon-Biet et al. 2014). When *M. musculus* were exposed to fasting in DR studies, their lifespan increased, while mice exposed to an *ad libitum* diet with nondigestible fiber did not have an increased lifespan (Pak et al. 2021; Solon-Biet et al. 2014). These observations are consistent with our results showing that flies exposed to non-fasting, but reduced nutrient intake, using indigestible cellulose for dilution, had reduced lifespans. However, Carey et al. (2022) found an increase in *D. melanogaster* lifespan with DR also using an *ad libitum* feeding and calorie restriction by diluting with indigestible cellulose. Furthermore, Lenhart et al. (2024) found that the effects of fasting in *D. melanogaster* depend on genetic background and do not always confer positive effects. These contrasting findings suggest complex and genotype-specific lifespan responses to diet.

The healthspan-related traits assessed in our study (dry weight, locomotor activity and HKDT) showed mixed results when exposed to different diets. When flies were exposed to a restricted diet, mean dry weight decreased (Figs. 2A and S2A, B, and Tables 2, S7 and S10), locomotor activity increased (Figs. 2B and S2C, D, and Tables 2, S8 and S13), and the directional change in HKDT depended on the age of the flies, with 7 day-old flies having higher and 16 days-old flies having lower HKDT (Figs. 2C and S3E, F, and Tables 2, S9 and S14). These effects of diet on healthspan metrics and lifespan show that aging is a complex and context-dependent phenomenon. Our finding that dietary nutritional conditions can have opposite, and trait- and genotype-specific impacts on lifespan and healthspan challenge our ability to make general recommendations as to which conditions are most favorable.

In our study, we investigated the effects of reduced nutrient intake on *D. melanogaster* at two specific ages: 7 days and 16 days. Although 16 days may not correspond to advanced aging in wildtype *D. melanogaster*, these ages were selected to investigate the effects of reduced nutrient intake during early adulthood, and for practical reasons (it turned out to be difficult to get enough flies surviving longer than 16 days on both diets). This choice allows us to capture the initial physiological responses to dietary interventions and the early onset of age-related changes. Notably, in the highly inbred DGRP system, lifespan varies significantly between lines, ranging from 16 to 71 days, with an average of 42 days under the environmental conditions used in Huang et al. (2020). This variability underscores the importance of examining the effects of reduced nutrient intake at different stages of early adulthood. We recognize that the impact of reduced nutrient intake can be influenced by both age and genetic background. To gain a more comprehensive understanding of the long-term effects of reduced nutrient intake, future studies should include older flies. Such an extension would provide deeper insights into how reduced nutrient intake interventions affect *D. melanogaster* across later life stages and genetic contexts.

Our study aimed to investigate trait variation in flies under different dietary conditions, focusing on the genetic background of DGRP lines. The $\hat{{H}^{2}}$ of lifespan was 0.39 on the control diet and 0.53 on the restricted diet (Figs. 4 and 5, and Tables S15 and S16), which are comparable to previous *D. melanogaster* studies ($\hat{{H}^{2}}=0.29-0.42$) (Durham et al. 2014; Huang et al. 2020; Ivanov et al. 2015). Dry weight $\hat{{H}^{2}}$ ranged from 0.25 to 0.34, aligning with the DGRP study by Jumbo-Lucioni et al. (2010), but lower than in Watanabe and Riddle (2021) who found $\hat{{H}^{2}}\approx 0.77$. Locomotor activity $\hat{{H}^{2}}$ ranged from 0.37 to 0.43, consistent with other DGRP studies ($\hat{{H}^{2}}=0.19-0.62$) (Jordan et al. 2007; Noer et al. 2024; Rohde et al. 2018; Videlier et al. 2021). HKDT $\hat{{H}^{2}}$ was between 0.12 and 0.36, also comparable to other DGRP findings ($\hat{{H}^{2}}=0.14-0.61$) (Lecheta et al. 2020; Rolandi et al. 2018; Soto et al. 2025). This suggests considerable genetic variation for all the traits. While it may appear that the heritability of lifespan is higher on the restricted diet compared to the control, it is important to note that the 95% confidence intervals of these heritabilities overlap. Although we can confirm that the heritabilities are different from zero, we cannot conclude that they are significantly different from each other due to this overlap. Therefore, it is not possible to assert that genetic variation plays a greater role for explaining variation in phenotype between lines on a restricted diet based on our data.

Environmental factors can influence genetic variation through environment-dependent gene action. This means that genes affecting a particular trait in one environment might not affect that trait in another environment. This phenomenon is often reflected in altered genetic correlations, either between different traits within a single environment or the same trait across various environments (Agrawal and Stinchcombe 2009; Sgrò and Hoffmann 2004; Vieira et al. 2000). Our findings indicate that the genetic correlations for dry weight, locomotor activity, and HKDT remain consistent across ages, suggesting that the same genes influence these traits across age-classes (Fig. 4 and Tables S15 and S16). However, the magnitude of these genetic correlations varies with diet. Specifically, the genetic correlations for dry weight and HKDT decrease when shifting from a control diet to a restricted diet, whereas the genetic correlation for locomotor activity increases under the same dietary change. This suggests that different regulatory pathways influence dry weight, locomotor activity, and HKDT depending on the diet. The pattern of change in genetic correlations across different diets is further validated by the highly significant GDI values for all traits (Tables S7–S9). The significance of GDI is mainly attributed to changes in the rank order of lines, rather than the magnitude of between-line genetic variance for all traits. Additionally, the GAI values were also significant for all traits (Tables S7–S9). The significance of GAI is primarily due to changes in the rank order of lines, rather than the magnitude of between-line genetic variance for all traits (Fig. S4 and Table S17). This indicates that trait values for dry weight, locomotor activity, HKDT, and lifespan in flies depend on their genetic background and age. These GDI and GAI findings underscore that dietary responses vary significantly not only among individuals with different genetic backgrounds, as shown previously (Francis et al. 2021; Huang et al. 2020; Patel and Talbert 2021), but also across different ages within those genetic backgrounds. This indicates that the effectiveness of dietary interventions depends on age, highlighting the importance of considering both genetic background and age when assessing dietary impacts on an individual.

Based on the overall mean lifespan of the DGRP population, the restricted diet reduced average lifespan compared to that on the control diet. This is not consistent with previous observations indicating that reduced nutrient intake is associated with increased lifespan (Durham et al. 2014; Li et al. 2024; Wilson et al. 2020). This could be due to malnutrition under the restricted diet in our study. However, we did observe significant genotype-by-diet and genotype-by-age interactions: while many genotypes experience decreased health and lifespan when exposed to the diluted diet, others exhibit increased health- and lifespan under the restricted diet. Hence, the effects of the restricted diet depends on the genetic background. Extrapolating to humans, this suggests that dietary interventions should be personalized and may need to be adjusted continuously throughout an individual’s life.

We conducted a GWAS to identify genetic variants associated with dietary response of lifespan, as well as age and diet response for dry weight, locomotor activity, and HKDT (Figs. 7 and S6–S9, and Tables S18 and S19). We identified a variant associated with genotype-by-diet interaction of dry weight of 7 day-old flies (Figs. 7A, B and Table S18), located at the intronic *PGRP-LC* locus. *PGRP-LC* encodes a transmembrane protein that acts as a pattern recognition receptor, identifying peptidoglycan in bacterial cell walls (Kaneko et al. 2006). It influences cellular immune responses by activating blood cells and enhancing their circulation independently of the Relish pathway, thereby playing a role in both humoral and cellular immunity (Borge-Renberg 2008). Activation of *PGRP-LC* in the IMD pathway in the fat body leads to the production of antimicrobial peptides during infections (Aggarwal and Silverman 2008). However, findings in the literature do not indicate that *PGRP-LC* directly regulates fat body metabolism or functions beyond its immune response role. Furthermore, *PGRP-LC* has been found to regulate lifespan but in a temperature-dependent manner in the DGRP (Huang et al. 2020). This suggests that *PGRP-LC* is important for immune regulation rather than metabolic functions in the fat body (Gendrin et al. 2013).

We also identified a SNP for the genotype-by-diet interaction affecting the lifespan of *D. melanogaster* (Figs. 7C, D and Table S19) at the *mbl* locus. The *mbl* gene encodes an RNA-binding protein crucial for RNA metabolism, including alternative splicing and microRNA (miRNA) biogenesis (Fernandez-Costa et al. 2011; Li and Millard 2019; Oddo et al. 2016). It is essential for muscle, eye, and neural tissue differentiation and plays a significant role in cardiac function, influencing lifespan in *D. melanogaster* (Li and Millard 2019; Mutsuddi et al. 2004). Overexpression of *mbl* in myotonic dystrophy type 1 (*DM1*) models improved muscle function and lifespan (Cerro-Herreros et al. 2016). Manipulating expression of *mbl* and its counterpart *Bru-3* led to asynchronous heartbeats and dilated cardiomyopathy, highlighting the role of *mbl* in cardiac health (Auxerre-Plantié et al. 2019; Souidi et al. 2023). These findings suggest that the regulatory functions of *mlb* in RNA processing may impact lifespan and aging in a diet-dependent manner. The candidate genes have only been identified in genotype-by-diet interactions, highlighting the crucial role of genetic variation in shaping the aging response to diet.

The GWAS results presented here indicate two candidate genes, *PGRP-LC* and *mbl*, which can be targeted for further research on aging. The *PGRP-LC* gene has a human ortholog, *PGLYRP1*, that encodes a peptidoglycan recognition protein involved in microbial defense and broader immune regulation. Beyond its antimicrobial functions, *PGLYRP1* has been shown to activate cytotoxic CD4+ and CD8 + T cells (Sharapova et al. 2018). The gene has also been implicated in rheumatoid arthritis, where elevated serum levels correlate with disease markers such as rheumatoid factor and anti-citrullinated protein antibodies (Luo et al. 2018). These findings suggest that *PGLYRP1* may serve as a biomarker and functional contributor to autoimmune pathogenesis. Moreover, *PGLYRP1* has emerged as a context-dependent immune regulator (Sharapova et al. 2017). Sharapova et al. (2017) showed that its deletion enhances anti-tumor immunity while simultaneously reducing autoimmune pathology in models of multiple sclerosis. This dual role, promoting tumor clearance via CD8 + T cells and limiting inflammation through myeloid cell modulation, highlights its potential as a therapeutic target of relevance in relation to both cancer and autoimmunity. Although not directly linked to body weight in humans, its role in systemic inflammation supports a plausible connection between immune signaling and metabolic traits, aligning with the function of *PGRP-LC* in flies. In addition, we identified *mbl*, *the D. melanogaster ortholog* of the human *MBNL2*, as being significantly associated with lifespan. *MBNL2* is an RNA-binding protein that regulates alternative splicing and plays a key role in stem cell differentiation, tissue regeneration, and tumor suppression (Lee et al. 2016). It is upregulated during liver regeneration and suppresses stemness-associated transcription factors such as *SOX2* and *NANOG*. In cancer models, *MBNL2* acts as a tumor suppressor, limiting proliferation and invasiveness, while its expression declines in more aggressive tumors. These functions are directly relevant to aging, as maintaining regenerative capacity and cellular homeostasis is essential for longevity.

While our current study focuses on mapping genetic variants, future research could employ allele swapping and knockdown experiments to further elucidate the functions of *PGRP-LC* and *mbl*. Specifically, allele swapping using CRISPR-Cas9 could be used to replace a mutant *PGRP-LC* allele with the wildtype allele to observe dry weight changes in dietary response (Gratz et al. 2015). Similarly, replacing a mutant *mbl* allele with the wildtype allele could determine if this rescues the flies by prolonging lifespan. Knockdown experiments using RNA interference (RNAi) could reduce *PGRP-LC* and *mbl* expression, allowing assessment of their respective impacts on dry weight and lifespan (Heigwer et al. 2018).

While our primary aim was to assess lifespan and healthspan through traits such as locomotor activity, dry weight, and heat knockdown time, we acknowledge that fecundity referring to the reproductive capacity of an organism, is crucial for understanding Darwinian fitness as it directly impacts the ability of a population to sustain and propagate. Including fecundity in future studies would provide insights into the trade-offs between reproductive fitness and healthspan traits. This would allow for a more comprehensive assessment of how dietary interventions affect both the longevity and reproductive success of *D. melanogaster*, thereby offering a holistic view of biological fitness.

## Supplementary information

Supplementary material 1

Supplementary material 2
