# Supplementary material for: The role of genetic variation in shaping phenotypic responses to diet in aging Drosophila melanogaster
Source: Heredity (Edinb). 2025 Sep 24;134(10-11):611–23. doi: 10.1038/s41437-025-00797-3 (PMC12594875; doi:10.1038/s41437-025-00797-3)
Supplement: Supplementary file 1 — Supplementary material 1 [file 41437_2025_797_MOESM1_ESM.pdf]

SUPPLEMENTARY MATERIAL FOR

The Role of Genetic Variation in Shaping Phenotypic Responses to Diet in Aging *Drosophila melanogaster*

This supplementary file contains:

Supplementary Figures

Figure S1	Correlations of estimated heritability between all data and balanced data
Figure S2	Histogram plots
Figure S3	Survival analysis of lifespan
Figure S4	GAI and GDI due to differences in magnitude of between line variance and changes in rank order
Figure S5	Reaction norm plots across ages
Figure S6	Q-Q plot and Manhattan plot for dry weight
Figure S7	Q-Q plot and Manhattan plot for locomotor activity
Figure S8	Q-Q plot and Manhattan plot for HKDT
Figure S9	Q-Q plot and Manhattan plot for lifespan

Supplementary Tables

Table S1	Number of flies and lines on balanced and unbalanced data of dry weight
Table S2	Number of flies and lines on balanced and unbalanced data of locomotor activity
Table S3	Number of flies and lines on balanced and unbalanced data of HKDT
Table S4	Summary of ANOVA on data of dry weight
Table S5	Summary of ANOVA on data of locomotor activity
Table S6	Summary of ANOVA on data of HKDT
Table S7	Summary of ANOVA on balanced data of dry weight
Table S8	Summary of ANOVA on balanced data of locomotor activity
Table S9	Summary of ANOVA on balanced data of HKDT
Table S10	Line mean and standard error of dry weight for separate days and diets [**Excel**]
Table S11	Summary of ANOVA on data of lifespan
Table S12	Line mean and standard error of lifespan for separate days and diets [**Excel**]
Table S13	Line mean and standard error of locomotor activity for separate days and diets [**Excel**]
Table S14	Line mean and standard error of HKDT for separate days and diets [**Excel**]
Table S15	Phenotypic correlation, heritability and genotypic correlation matrix of all traits with standard errors [**Excel**]
Table S16	Phenotypic correlation, heritability and genotypic correlation matrix of all traits with 95% Confidence intervals [**Excel**]
Table S17	Matrix of the percent contribution of changes in rank order of all traits [**Excel**]
Table S18	GWAS results for the genotype-by-diet interaction for dry weight of 7 day-old flies [**Excel**]
Table S19	GWAS results for the genotype-by-diet interaction for lifespan of flies [**Excel**]

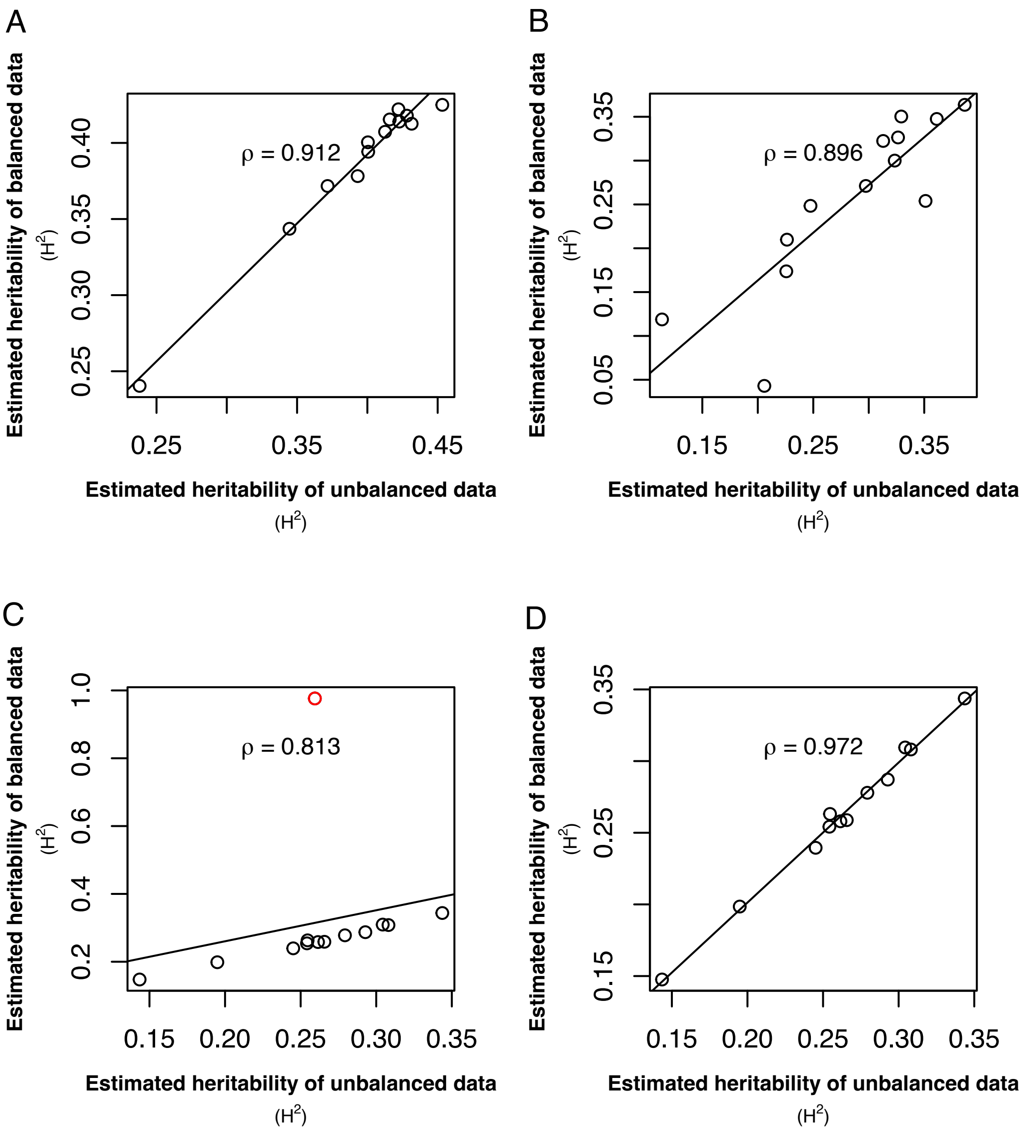

Figure S1. Correlations of estimated heritability between all data and unbalanced data. The balanced dataset was generated by excluding DGRP lines that were not present in both control and restricted diets, whereas the unbalanced dataset included all lines. The figures represent healthspan metrics: (**A**) locomotor activity, (**B**) heat knockdown time (HKDT), (**C**) dry weight with an outlier indicated as a red point, and (**D**) dry weight excluding the outlier. The symbol 'ρ' denotes the Spearman correlation coefficient.

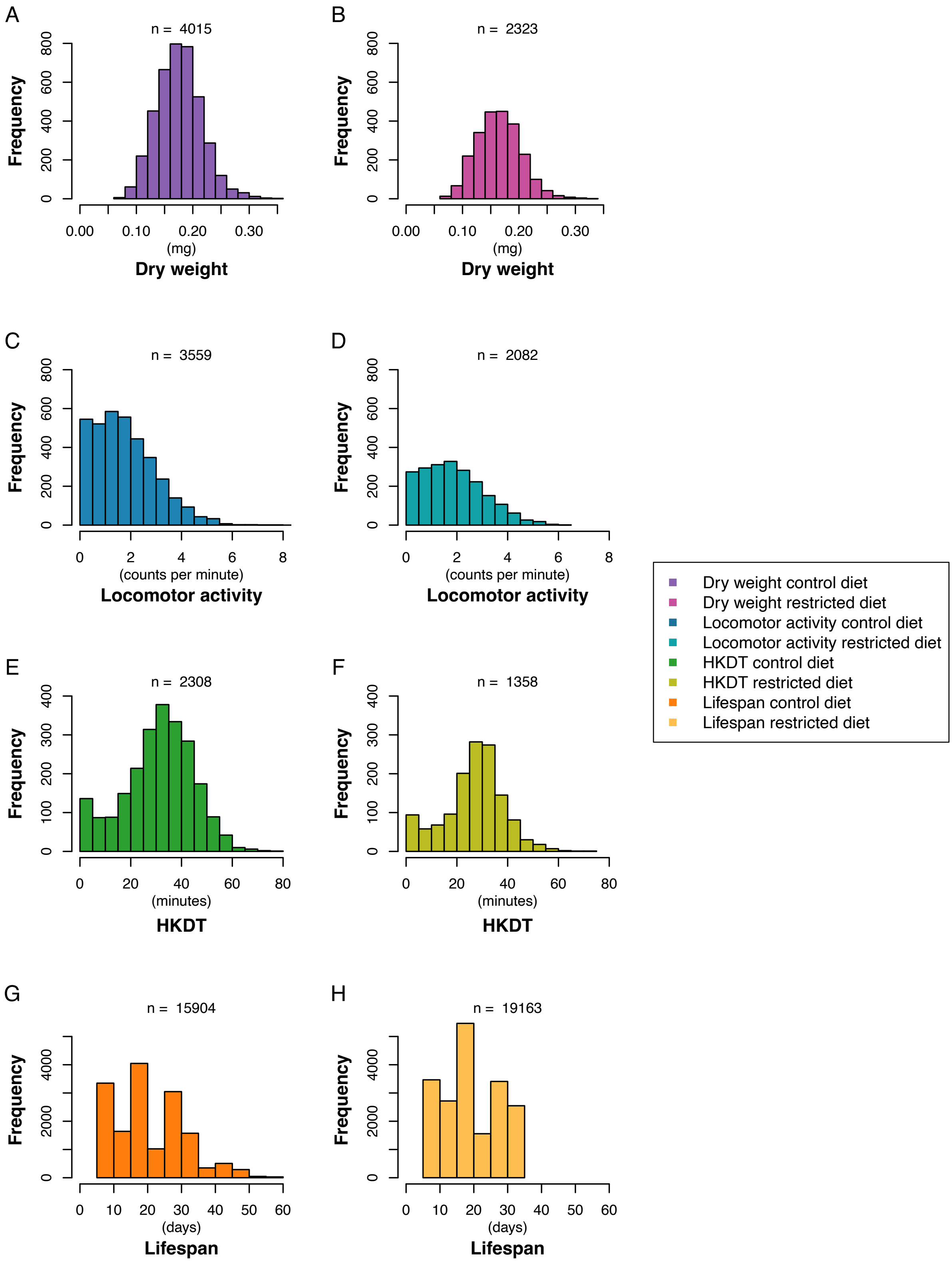

Figure S2. Histogram plots. Histograms for (**A**,**B**) dry weight (purple), (**C**,**D**) locomotor activity (blue), (**E**,**F**) HKDT (green) and (**G**,**H**) lifespan (orange) on control (dark shades) and restricted (light shades) diets.

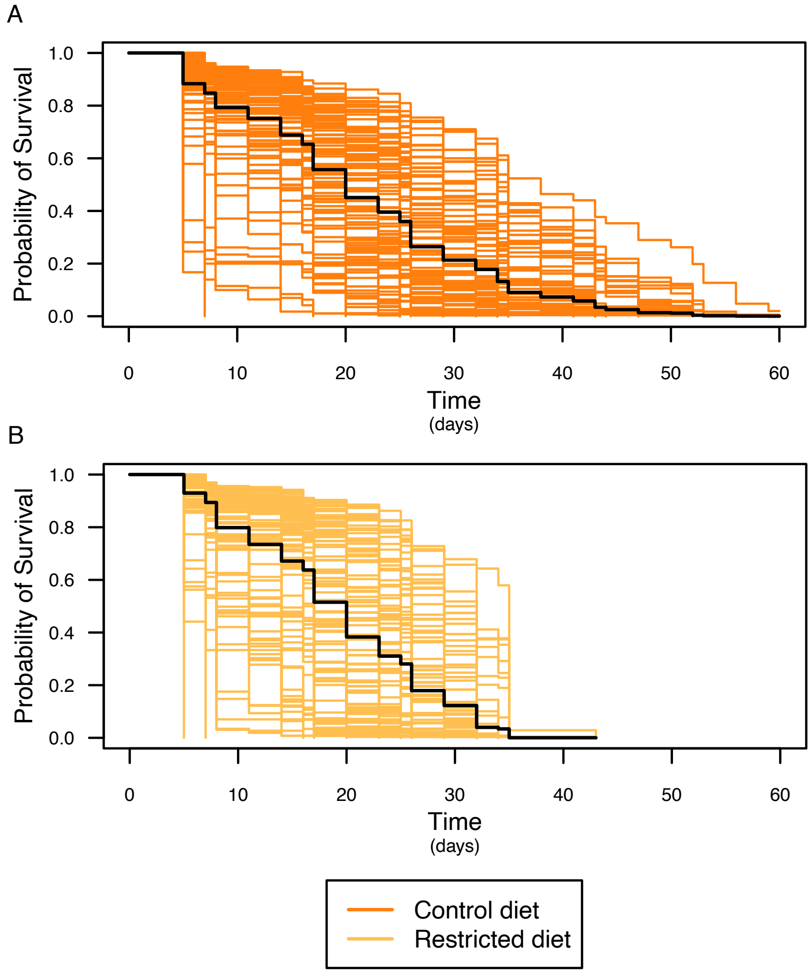

Figure S3. Survival analysis of lifespan. Lines fed on (**A**) control diet (dark orange), (**B**) restricted diet (light orange), where black lines indicate mean lifespan for separate diets.

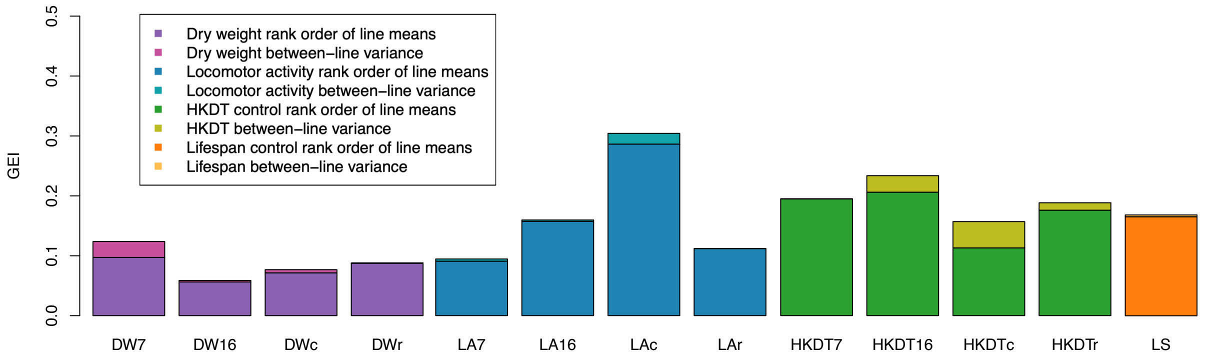

Figure S4. GAI and GDI due to differences in magnitude of between line variance and changes in rank order. The relative proportion of interaction variance due to these effects for genotype-by-diet or genotype-by-age interactions for dry weight (DW), lifespan (LS), heat knockdown time (HKDT), and locomotor activity (LA). The age groups are 7 day-old (7) and 16 day-old (16) flies, and the diet types are control (c) and restricted (r) diets. Colors indicate the proportion of GDI and GAI attributed by differences in magnitude of between-line variance (light shades) of genetic variance between the control and restricted diet or age 7 and 16 days and rank order of line means (dark shades).

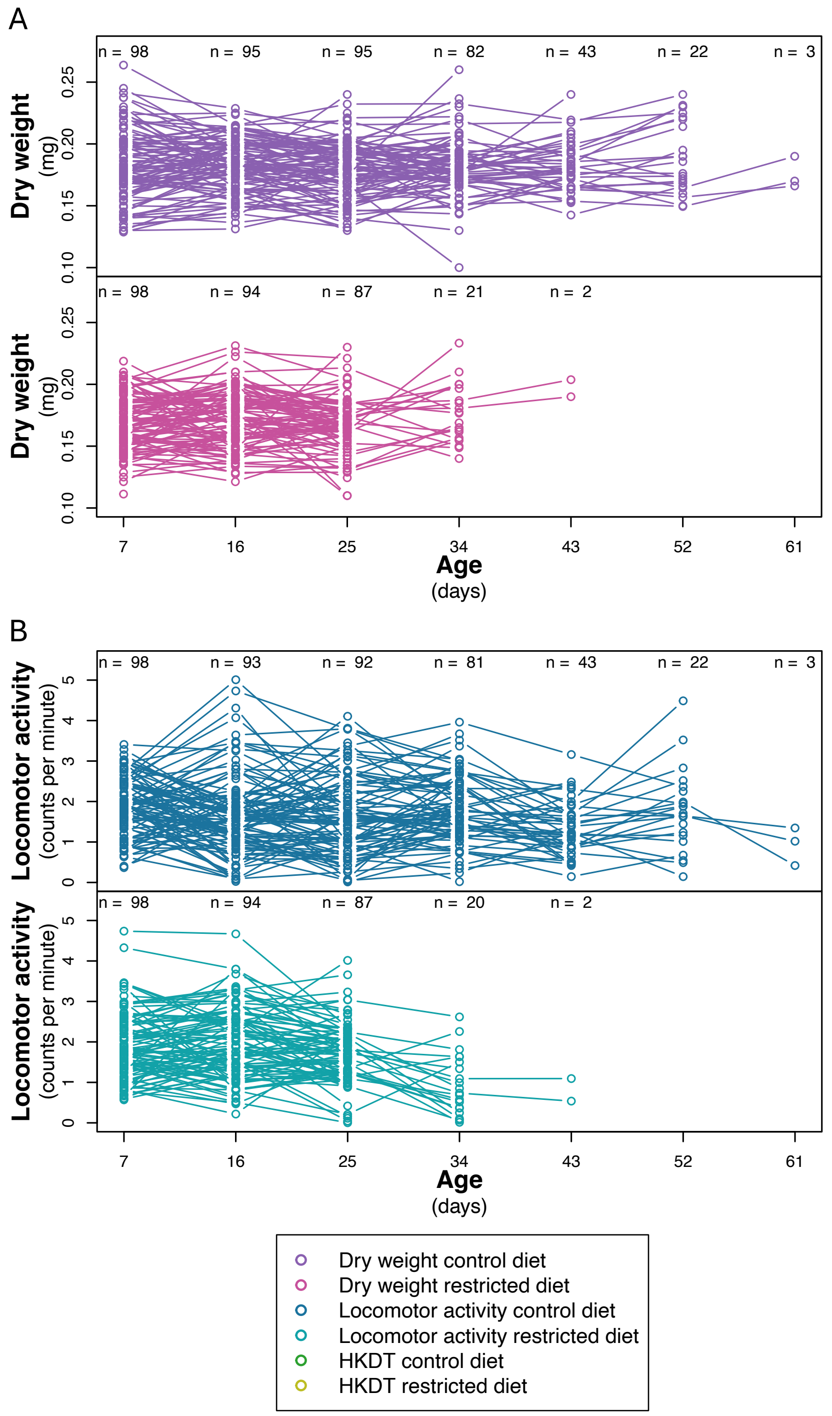

Figure S5. Reaction norm plots across ages. Line means of (**A**) dry weight (purple), (**B**) locomotor activity (blue) and (**C**) HKDT (green) across seven ages, fed on control diet (dark shades) and restricted diet (light shades). Points represent the mean phenotype of each DGRP line, and lines connecting points across age represent the same DGRP line. Numbers (n) of DGRP lines assessed at each age and diet are indicated above the points.

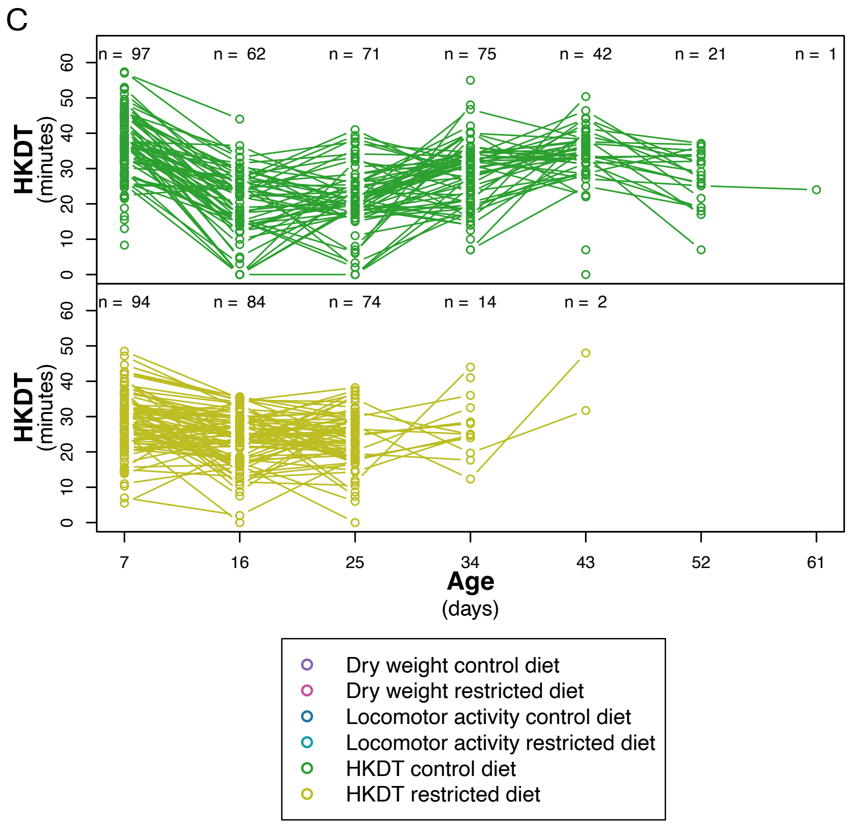

Figure S5. Reaction norm plots across ages (continued). Line means of (**A**) dry weight (purple), (**B**) locomotor activity (blue) and (**C**) HKDT (green) across seven ages, fed on control diet (dark shades) and restricted diet (light shades). Points represent the mean phenotype of each DGRP line, and lines connecting points across age represent the same DGRP line. Numbers (n) of DGRP lines assessed at each age and diet are indicated above the points.

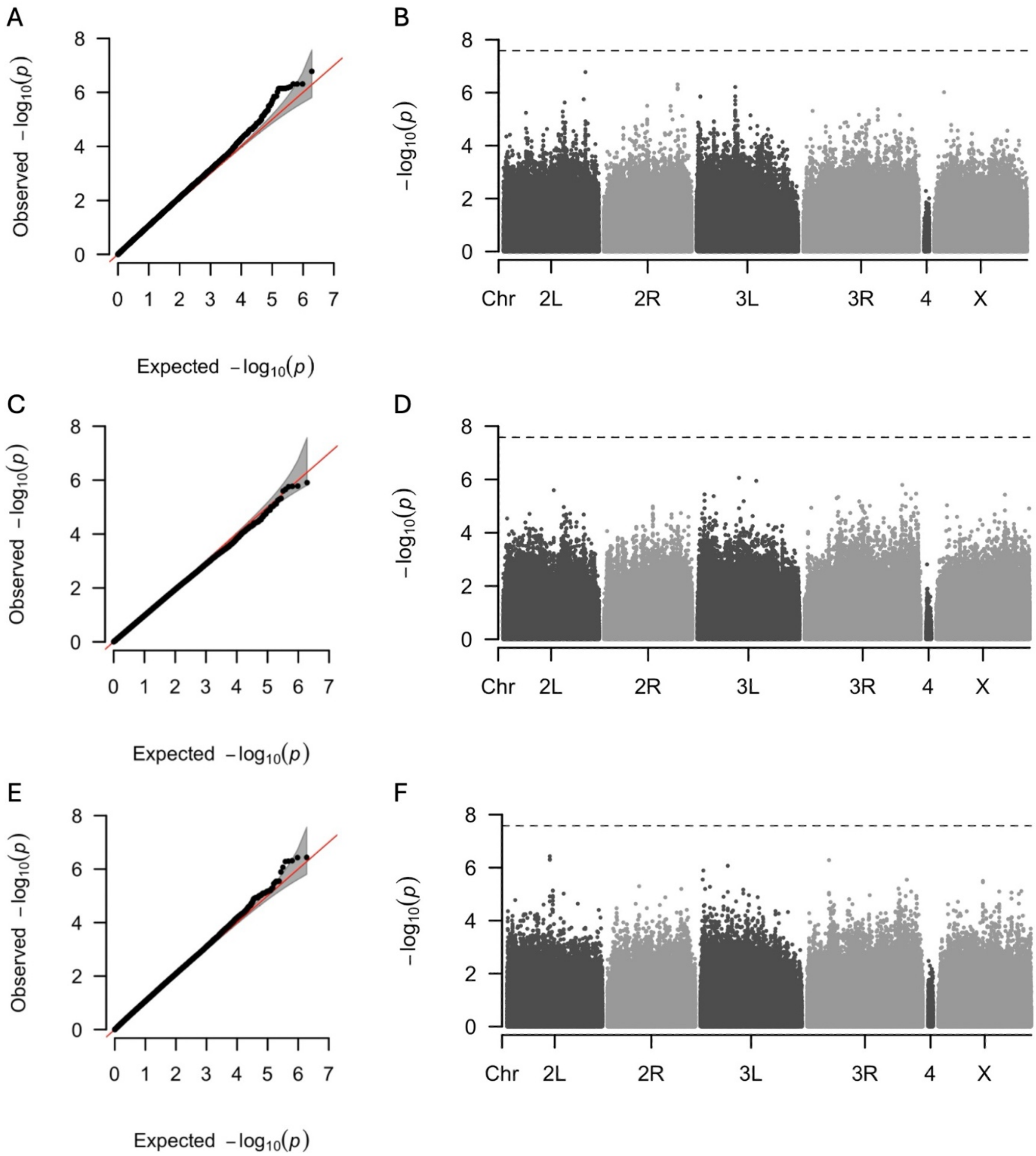

Figure S6. **Q-Q plot and Manhattan plot for dry weight.** Results of a GWAS to identify variants associated with dry weight of (**A**, **B**) 7 day-old flies fed the control diet, (**C**, **D**) 7 day-old flies fed the restricted diet, (**E**, **F**) 16 day-old flies fed the control diet, (**G**, **H**) 16 day-old flies fed the restricted diet, (**I**, **J**) genotype-by-diet interaction for 16 day-old flies, and (**K**, **L**) genotype-by-age interaction for flies fed the restricted diet. Panels (**A**, **C**, **E**, **G**, **I**, **K**) are Q-Q plots comparing the observed -log(*p*) values of each variant to the expected values, with the red line representing the null expectation and the grey area indicating the confidence interval. Panels (**B**, **D**, **F**, **H**, **J**, **L**) are Manhattan plots where each point represents a variant. The *y*-axis shows the strength of the association between individual variants and dry weight, expressed as -log(*p*). The dashed horizontal line indicates the significance threshold adjusted for multiple testing using the Bonferroni correction.

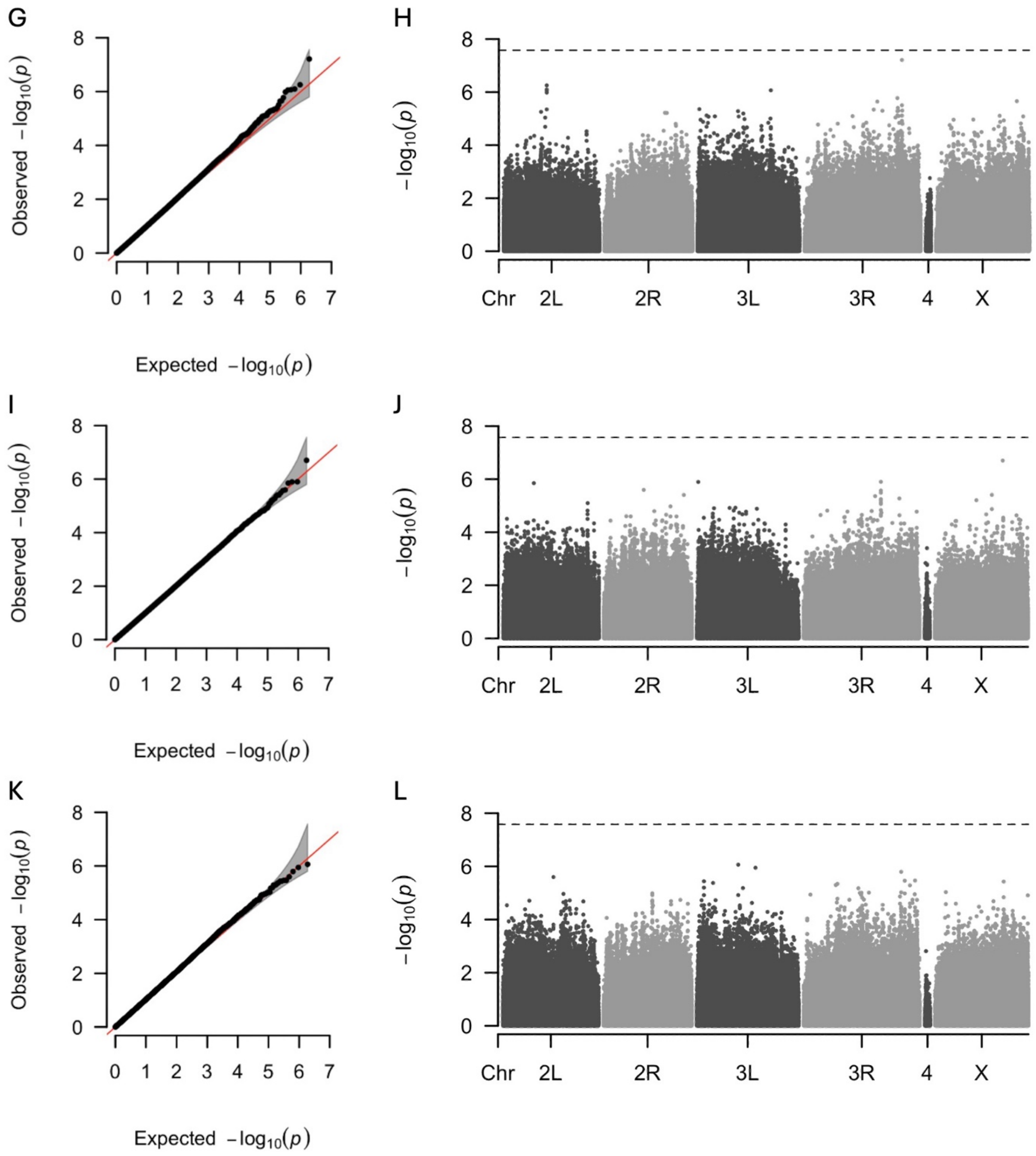

Figure S6 (continued). **Q-Q plot and Manhattan plot for dry weight.** Results of a GWAS to identify variants associated with dry weight of (**A**, **B**) 7 day-old flies fed the control diet, (**C**, **D**) 7 day-old flies fed the restricted diet, (**E**, **F**) 16 day-old flies fed the control diet, (**G**, **H**) 16 day-old flies fed the restricted diet, (**I**, **J**) genotype-by-diet interaction for 16 day-old flies, and (**K**, **L**) genotype-by-age interaction for flies fed the restricted diet. Panels (**A**, **C**, **E**, **G**, **I**, **K**) are Q-Q plots comparing the observed -log(*p*) values of each variant to the expected values, with the red line representing the null expectation and the grey area indicating the confidence interval. Panels (**B**, **D**, **F**, **H**, **J**, **L**) are Manhattan plots where each point represents a variant. The *y*-axis shows the strength of the association between individual variants and dry weight, expressed as -log(*p*). The dashed horizontal line indicates the significance threshold adjusted for multiple testing using the Bonferroni correction.

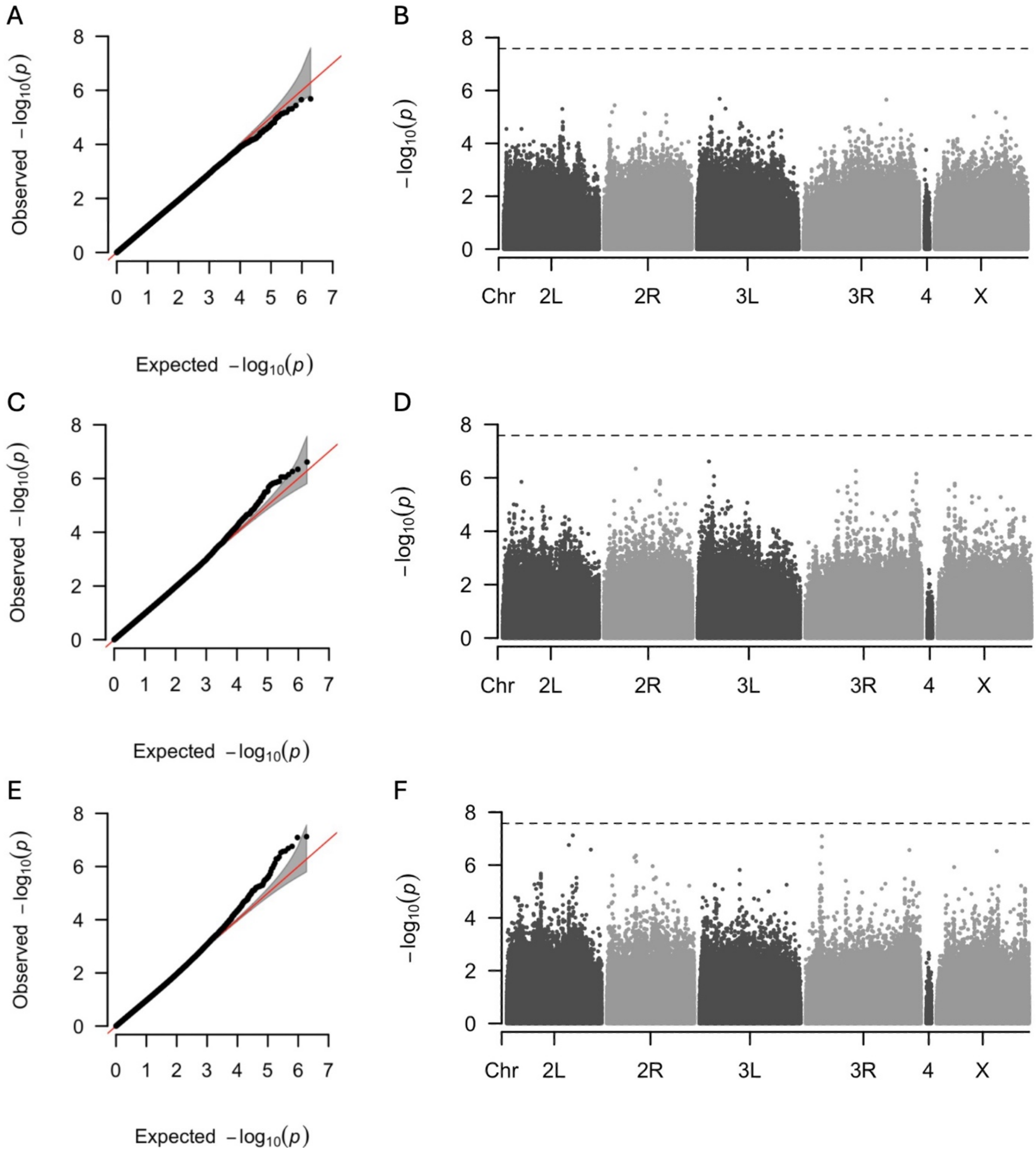

Figure S7. **Q-Q plot and Manhattan plot for locomotor activity.** Results of a GWAS to identify variants associated with locomotor activity of (**A**, **B**) 7 day-old flies fed the control diet, (**C**, **D**) 7 day-old flies fed the restricted diet, (**E**, **F**) 16 day-old flies fed the control diet, (**G**, **H**) 16 day-old flies fed the restricted diet, (**I**, **J**) genotype-by-diet interaction for 7 day-old flies, (**K**, **L**) genotype-by-diet interaction for 16 day-old flies, (**M**, **N**) genotype-by-age interaction for flies fed the control diet, and (**O**, **P**) genotype-by-age interaction for flies fed the restricted diet. Panels (**A**, **C**, **E**, **G**, **I**, **K**, **M**, **O**) are Q-Q plots comparing the observed -log(p) values of each variant to the expected values, with the red line representing the null expectation and the grey area indicating the confidence interval. Panels (**B**, **D**, **F**, **H**, **J**, **L**, **N**, **P**) are Manhattan plots where each point represents a variant. The y-axis shows the strength of the association between individual variants and dry weight, expressed as -log(p). The dashed horizontal line indicates the significance threshold adjusted for multiple testing using the Bonferroni correction.

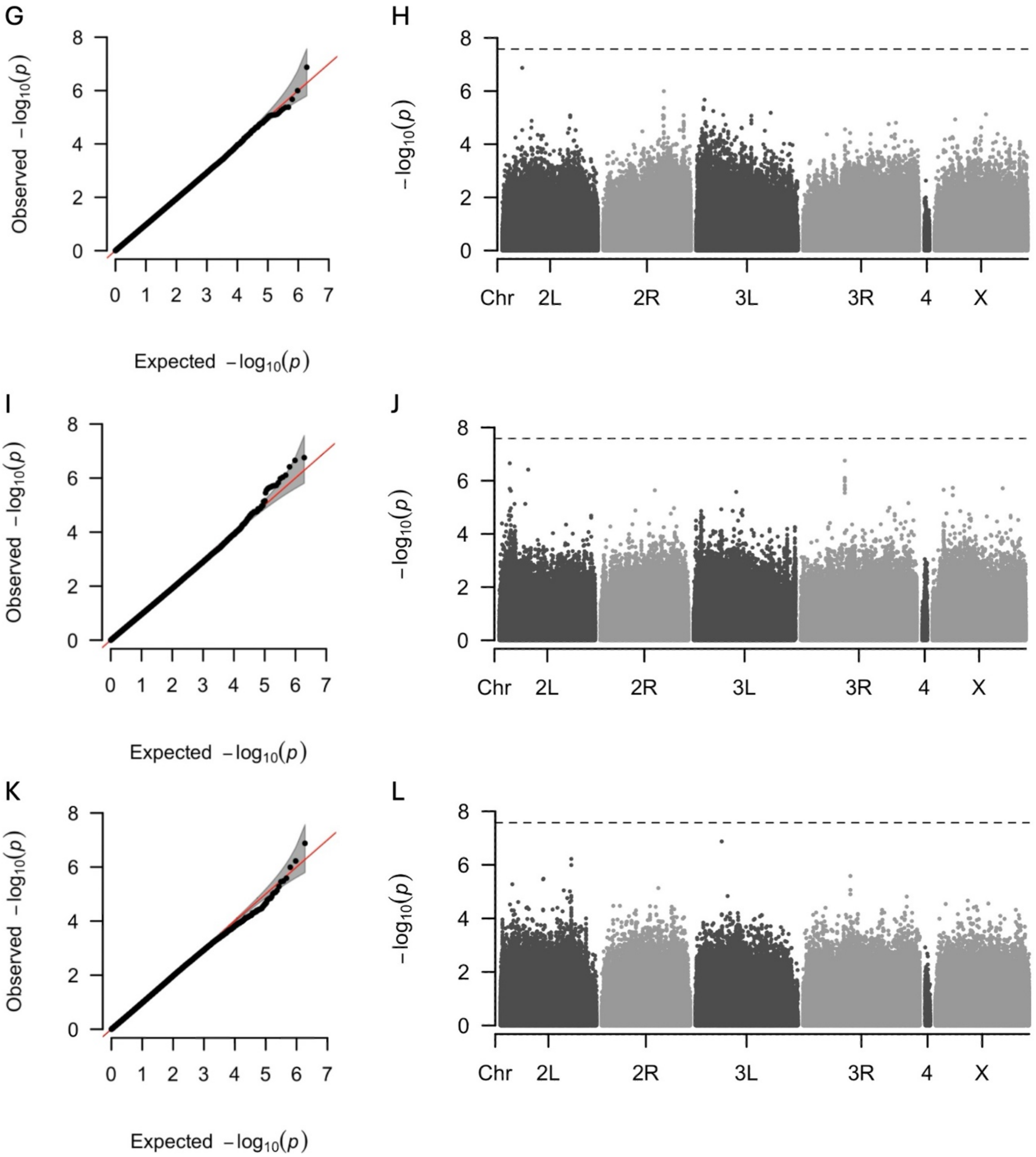

Figure S7 (continued). **Q-Q plot and Manhattan plot for locomotor activity.** Results of a GWAS to identify variants associated with locomotor activity of (**A**, **B**) 7 day-old flies fed the control diet, (**C**, **D**) 7 day-old flies fed the restricted diet, (**E**, **F**) 16 day-old flies fed the control diet, (**G**, **H**) 16 day-old flies fed the restricted diet, (**I**, **J**) genotype-by-diet interaction for 7 day-old flies, (**K**, **L**) genotype-by-diet interaction for 16 day-old flies, (**M**, **N**) genotype-by-age interaction for flies fed the control diet, and (**O**, **P**) genotype-by-age interaction for flies fed the restricted diet. Panels (**A**, **C**, **E**, **G**, **I**, **K, M, O**) are Q-Q plots comparing the observed -log(p) values of each variant to the expected values, with the red line representing the null expectation and the grey area indicating the confidence interval. Panels (**B**, **D**, **F**, **H**, **J**, **L**, **N, P**) are Manhattan plots where each point represents a variant. The y-axis shows the strength of the association between individual variants and dry weight, expressed as -log(p). The dashed horizontal line indicates the significance threshold adjusted for multiple testing using the Bonferroni correction.

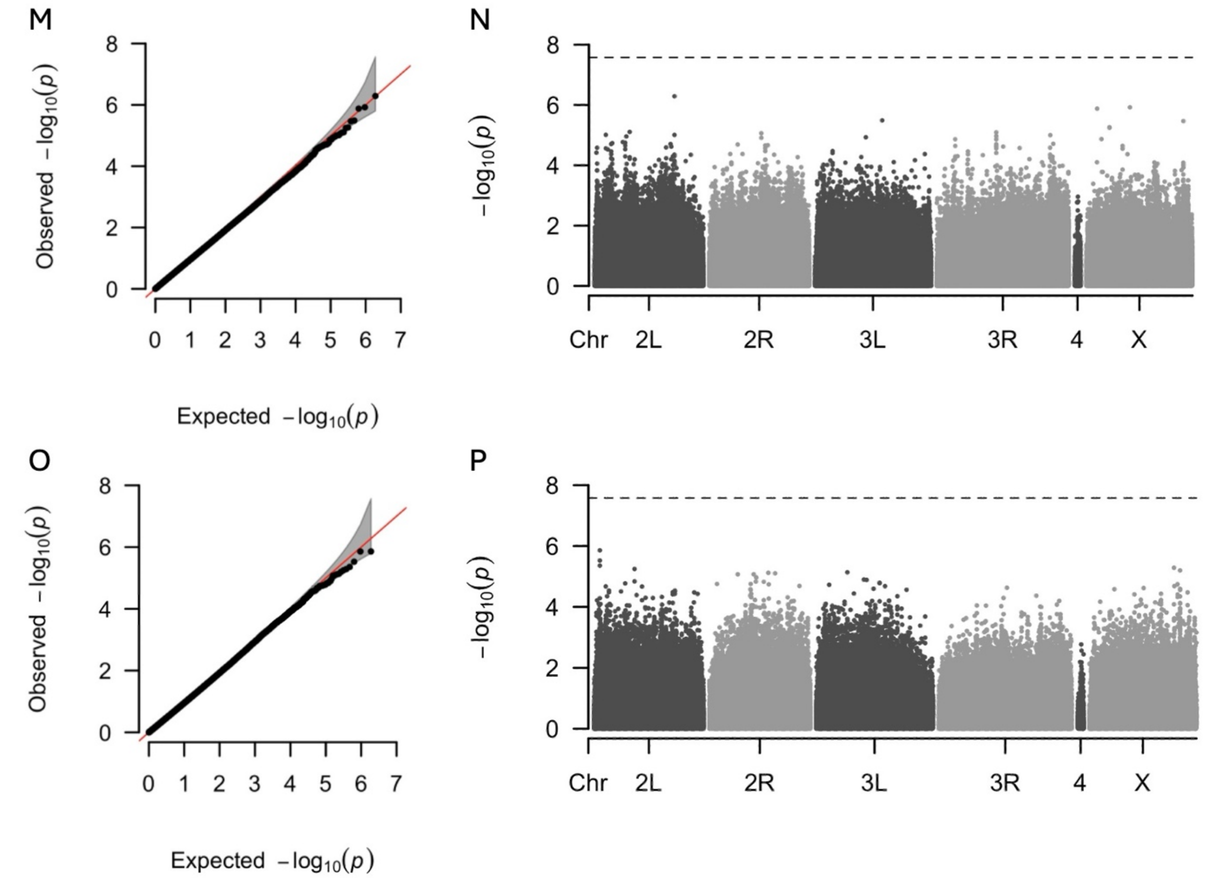

Figure S7 (continued). **Q-Q plot and Manhattan plot for locomotor activity.** Results of a GWAS to identify variants associated with locomotor activity of (**A**, **B**) 7 day-old flies fed the control diet, (**C**, **D**) 7 day-old flies fed the restricted diet, (**E**, **F**) 16 day-old flies fed the control diet, (**G**, **H**) 16 day-old flies fed the restricted diet, (**I**, **J**) genotype-by-diet interaction for 7 day-old flies, (**K**, **L**) genotype-by-diet interaction for 16 day-old flies, (**M**, **N**) genotype-by-age interaction for flies fed the control diet, and (**O**, **P**) genotype-by-age interaction for flies fed the restricted diet. Panels (**A**, **C**, **E**, **G**, **I**, **K, M, O**) are Q-Q plots comparing the observed -log(p) values of each variant to the expected values, with the red line representing the null expectation and the grey area indicating the confidence interval. Panels (**B**, **D**, **F**, **H**, **J**, **L, N, P**) are Manhattan plots where each point represents a variant. The y-axis shows the strength of the association between individual variants and dry weight, expressed as -log(p). The dashed horizontal line indicates the significance threshold adjusted for multiple testing using the Bonferroni correction.

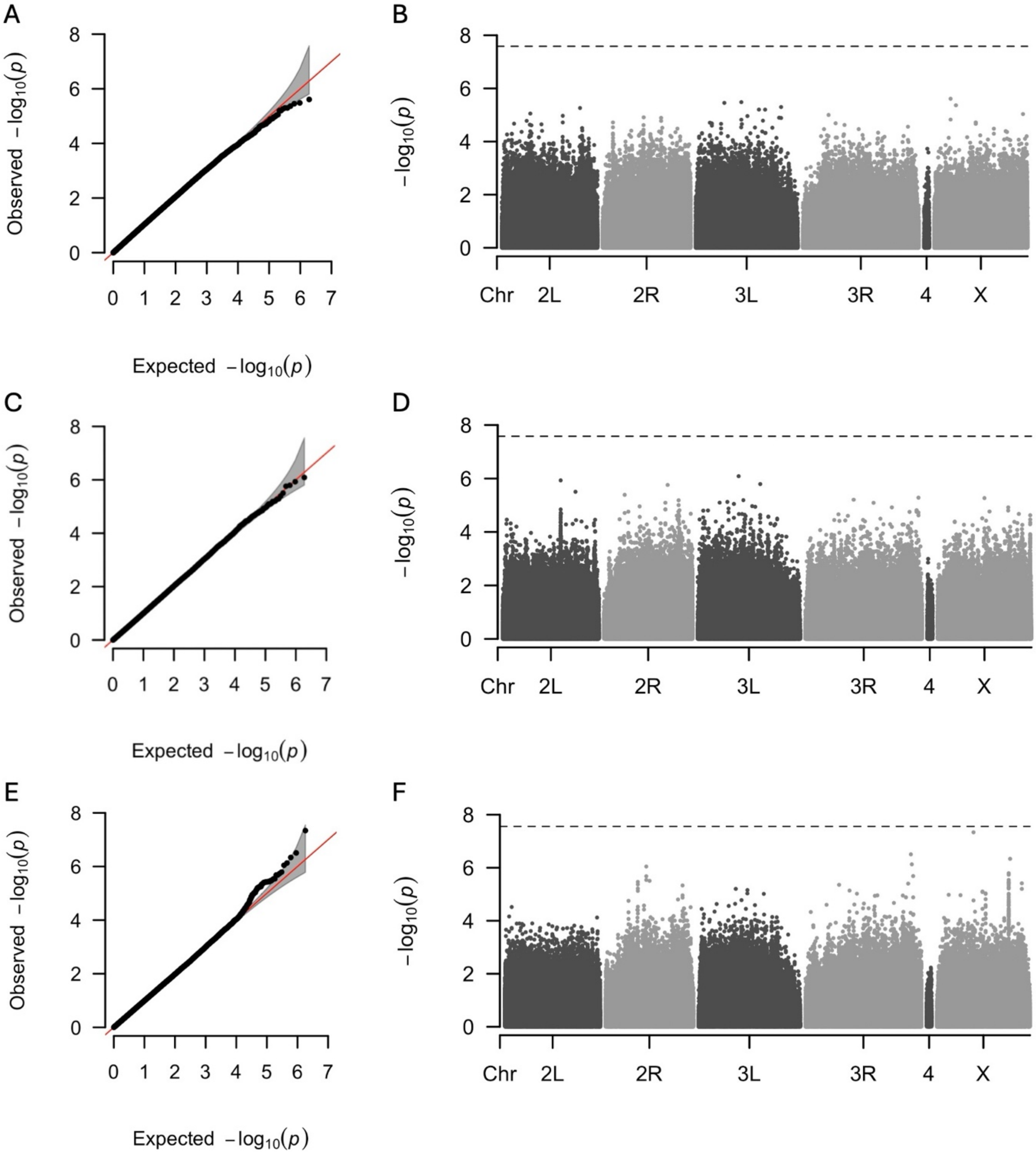

Figure S8. **Q-Q plot and Manhattan plot for HKDT.** Results of a GWAS to identify variants associated with HKDT of (**A**, **B**) 7 day-old flies fed the control diet, (**C**, **D**) 7 day-old flies fed the restricted diet, (**E**, **F**) 16 day-old flies fed the restricted diet, (**G**, **H**) genotype-by-diet interaction for 7 day-old flies, (**I**, **J**) genotype-by-diet interaction for 16 day-old flies, (**K**, **L**) genotype-by-age interaction for flies fed the control diet, and (**M**, **N**) genotype-by-age interaction for flies fed the restricted diet. Panels (**A**, **C**, **E**, **G**, **I**, **K, M**are Q-Q plots comparing the observed -log(p) values of each variant to the expected values, with the red line representing the null expectation and the grey area indicating the confidence interval. Panels (**B**, **D**, **F**, **H**, **J**, **L, N**) are Manhattan plots where each point represents a variant. The y-axis shows the strength of the association between individual variants and dry weight, expressed as -log(p). The dashed horizontal line indicates the significance threshold adjusted for multiple testing using the Bonferroni correction.

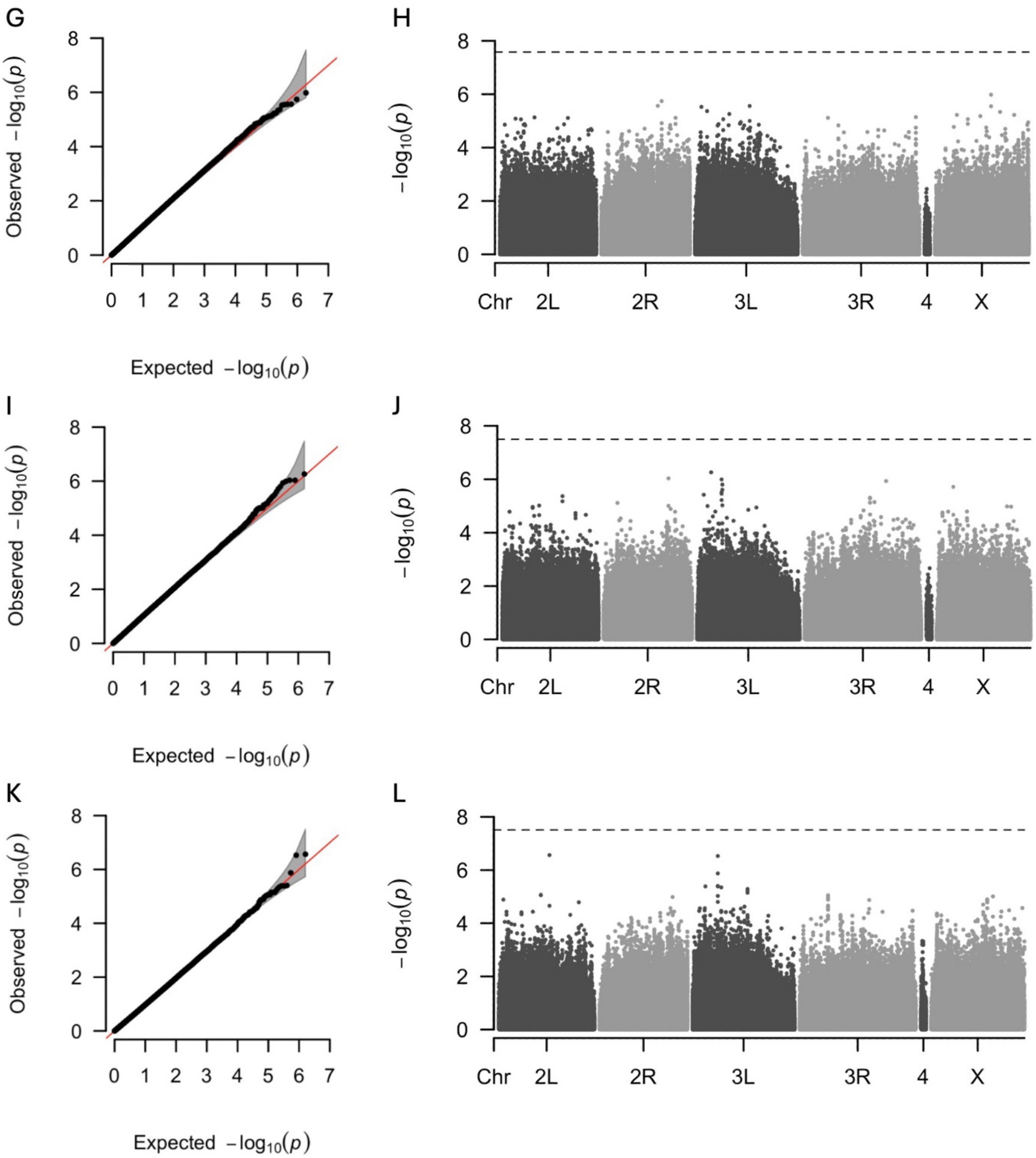

Figure S8 (continued). **Q-Q plot and Manhattan plot for HKDT.** Results of a GWAS to identify variants associated with HKDT of (**A**, **B**) 7 day-old flies fed the control diet, (**C**, **D**) 7 day-old flies fed the restricted diet, (**E**, **F**) 16 day-old flies fed the restricted diet, (**G**, **H**) genotype-by-diet interaction for 7 day-old flies, (**I**, **J**) genotype-by-diet interaction for 16 day-old flies, (**K**, **L**) genotype-by-age interaction for flies fed the control diet, and (**M**, **N**) genotype-by-age interaction for flies fed the restricted diet. Panels (**A**, **C**, **E**, **G**, **I**, **K, M**are Q-Q plots comparing the observed -log(p) values of each variant to the expected values, with the red line representing the null expectation and the grey area indicating the confidence interval. Panels (**B**, **D**, **F**, **H**, **J**, **L, N**) are Manhattan plots where each point represents a variant. The y-axis shows the strength of the association between individual variants and dry weight, expressed as -log(p). The dashed horizontal line indicates the significance threshold adjusted for multiple testing using the Bonferroni correction.

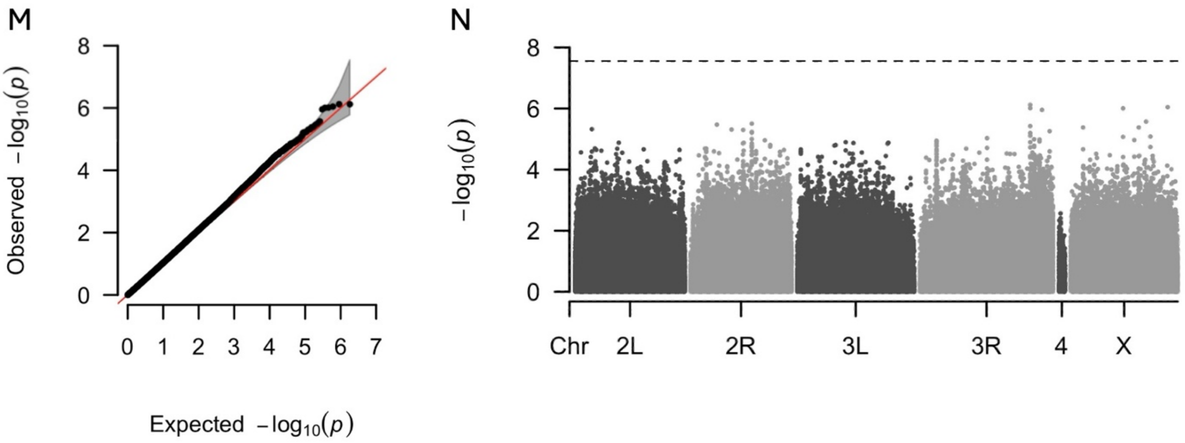

Figure S8 (continued). **Q-Q plot and Manhattan plot for HKDT.** Results of a GWAS to identify variants associated with HKDT of (**A**, **B**) 7 day-old flies fed the control diet, (**C**, **D**) 7 day-old flies fed the restricted diet, (**E**, **F**) 16 day-old flies fed the restricted diet, (**G**, **H**) genotype-by-diet interaction for 7 day-old flies, (**I**, **J**) genotype-by-diet interaction for 16 day-old flies, (**K**, **L**) genotype-by-age interaction for flies fed the control diet, and (**M**, **N**) genotype-by-age interaction for flies fed the restricted diet. Panels (**A**, **C**, **E**, **G**, **I**, **K**, **M**are Q-Q plots comparing the observed -log(p) values of each variant to the expected values, with the red line representing the null expectation and the grey area indicating the confidence interval. Panels (**B**, **D**, **F**, **H**, **J**, **L**, **N**) are Manhattan plots where each point represents a variant. The y-axis shows the strength of the association between individual variants and dry weight, expressed as -log(p). The dashed horizontal line indicates the significance threshold adjusted for multiple testing using the Bonferroni correction.

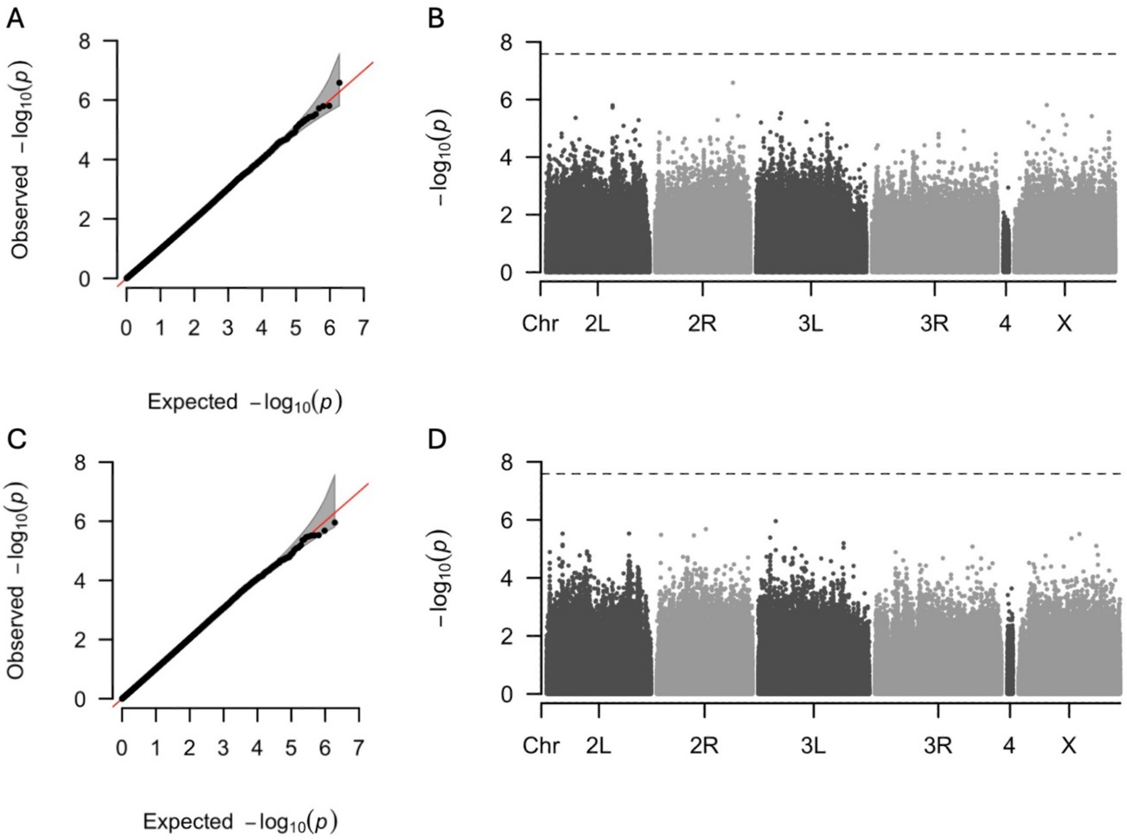

Figure S9. **Q-Q plot and Manhattan plot for lifespan.** Results of a GWAS to identify variants associated with lifespan of (**A**, **B**) flies fed the control diet and (**C**, **D**) flies fed the restricted diet. Panels (**A**, **C**) are Q-Q plots comparing the observed -

log(p) values of each variant to the expected values, with the red line representing the null expectation and the grey area indicating the confidence interval. Panels (**B**, **D**) are Manhattan plots where each point represents a variant. The y-axis shows the strength of the association between individual variants and dry weight, expressed as -log(p). The dashed horizontal line indicates the significance threshold adjusted for multiple testing using the Bonferroni correction.

Table S1. Number of flies across age groups and diets for both unbalanced and balanced datasets of dry weight measurements.

Type of test	Unbalanced		Balanced	
	Control diet	Restricted diet	Control diet	Restricted diet
Two-way age 1	778	783	778	783
Two-way age 2	756	748	740	740
Two-way age 3	785	655	718	642
Two-way age 4	998	128	308	128
Two-way age 5	521	9	32	9
Three-way age 1 and 2	1534	1531	1479	1483
Three-way age 1, 2 and 3	2319	2186	2030	1963
Three-way age 1, 2, 3 and 4	3317	2314	834	650

Table S2. Number of flies across age groups and diets for both unbalanced and balanced datasets of locomotor activity measurements.

Type of test	Unbalanced		Balanced	
	Control diet	Restricted diet	Control diet	Restricted diet
Two-way age 1	770	758	770	758
Two-way age 2	617	672	602	660
Two-way age 3	618	557	579	541
Two-way age 4	900	87	235	86
Two-way age 5	489	8	25	8
Three-way age 1 and 2	1387	1430	1320	1367
Three-way age 1, 2 and 3	2005	1987	1755	1749
Three-way age 1, 2, 3 and 4	2905	2074	623	481

Table S3. Number of flies across age groups and diets for both unbalanced and balanced datasets of HKDT measurements.

Type of test	Unbalanced		Balanced	
	Control diet	Restricted diet	Control diet	Restricted diet
Two-way age 1	691	558	669	558
Two-way age 2	225	430	214	322
Two-way age 3	228	320	257	261
Two-way age 4	553	43	105	39
Two-way age 5	430	8	17	8
Three-way age 1 and 2	916	988	645	696
Three-way age 1, 2 and 3	1204	1308	714	748
Three-way age 1, 2, 3 and 4	1757	1351	194	140

Table S4. Summary of full and reduced ANOVA models assessing dry weight of DGRP flies at 7, 16 and 25 days old and fed control and restricted diets. Diet and age were considered fixed effects, and DGRP line is a random effect.

Analysis	Source	DF	MS	F Value	*p*-value	σ^2	Lower	Upper	H^2
7 days old on control diet	L	97	6.578E-3	5.03	<0.0001	0.686E-3	0.442E-3	0.929E-3	0.3437
	Rep(L)	170	1.308E-3	1.00	0.4956	-0.594E-6	-0.11E-3	0.106E-3	
	ϵ	510	1.310E-3	-	-	1.310E-3	1.163E-3	0.149E-3	
7 days old on restricted diet	L	97	3.885E-3	2.96	<0.0001	0.332E-3	0.185E-3	0.478E-3	0.2542
	Rep(L)	169	1.267E-3	1.30	0.0155	0.107E-3	-3.03E-6	0.217E-3	
	ϵ	516	0.974E-3	-	-	0.974E-3	0.866E-3	1.105E-3	
7 days old on both diets	D	1	0.0761	26.61	<0.0001	Fixed	Fixed	Fixed	0.3081
	L	97	7.572E-3	2.63	<0.0001	0.304E-3	0.156E-3	0.452E-3	
	D×L	97	2.880E-3	2.20	<0.0001	0.204E-3	0.092E-3	0.315E-3	
	Rep(D×L)	339	1.288E-3	1.13	0.0823	0.054E-3	-0.02E-3	0.131E-3	
	ϵ	1026	1.141E-3	-	-	1.141E-3	1.049E-3	1.247E-3	
16 days old on control diet	L	94	3.643E-3	3.47	<0.0001	0.336E-3	0.196E-3	0.475E-3	0.2451
	Rep(L)	167	1.049E-3	1.01	0.4472	4.417E-6	-0.008E-3	0.093E-3	
	ϵ	494	1.035E-3	-	-	1.035E-3	0.917E-3	1.177E-3	

16 days old on restricted diet	L	93	4.386E-3	3.68	<0.0001	0.420E-3	0.252E-3	0.589E-3	0.2793
	Rep(L)	164	1.179E-3	1.09	0.2473	0.035E-3	-0.007E-3	0.137E-3	
	ϵ	490	1.084E-3	-	-	1.084E-3	0.960E-3	1.233E-3	
16 days old on both diets	D	1	0.0560	31.79	<0.0001	Fixed	Fixed	Fixed	0.2615
	L	95	6.134E-3	3.46	<0.0001	0.290E-3	0.169E-3	0.412E-3	
	D×L	92	1.770E-3	1.58	0.0026	0.085E-3	0.015E-3	0.115E-3	
	Rep(D×L)	331	1.113E-3	1.05	0.2832	0.020E-3	-0.005E-3	0.085E-3	
	ϵ	984	1.059E-3	-	-	1.059E-3	0.971E-3	1.159E-3	
7 and 16 days old on control diet	A	1	0.949E-3	0.45	0.5036	Fixed	Fixed	Fixed	0.3043
	L	97	8.058E-3	3.82	<0.0001	0.393E-3	0.236E-3	0.549E-3	
	A×L	94	2.110E-3	1.79	0.0002	0.121E-3	0.038E-3	0.203E-3	
	Rep(A×L)	337	1.180E-3	1.00	0.4731	2.039E-6	-0.006E-3	0.069E-3	
	ϵ	1004	1.175E-3	-	-	1.175E-3	1.078E-3	1.284E-3	
7 and 16 days old on restricted diet	A	1	2.430E-3	1.12	0.2929	Fixed	Fixed	Fixed	0.2657
	L	97	5.933E-3	2.69	<0.0001	0.248E-3	0.128E-3	0.369E-3	
	A×L	93	2.194E-3	1.76	0.0002	0.124E-3	0.042E-3	0.205E-3	
	Rep(A×L)	333	1.223E-3	1.19	0.0232	0.072E-3	-3.7E-6	0.148E-3	
	ϵ	1006	1.028E-3	-	-	1.028E-3	0.943E-3	1.124E-3	
7 and 16 days old on both diets	A	1	2.748E-3	1.35	0.2489	Fixed	Fixed	Fixed	0.2928
	D	1	0.1362	57.72	<0.0001	Fixed	Fixed	Fixed	
	L	97	0.0116	5.41	<0.0001	0.314E-3	0.199E-3	0.429E-3	
	A×D	1	0.191E-3	0.08	0.7723	Fixed	Fixed	Fixed	
	A×L	95	2.048E-3	0.90	0.6941	-0.002E-3	-0.07E-3	0.041E-3	
	D×L	97	2.368E-3	1.04	0.4271	5.948E-6	-0.06E-3	0.068E-3	
	A×D×L	92	2.275E-3	1.88	<0.0001	0.138E-3	0.050E-3	0.227E-3	
	Rep(A×D×L)	670	1.202E-3	1.09	0.0800	0.037E-3	-0.01E-3	0.087E-3	
	ϵ	2010	1.101E-3	-	-	1.101E-3	1.036E-3	1.172E-3	
25 days old on control diet	L	94	3.751E-3	2.87	<0.0001	0.306E-3	0.165E-3	0.448E-3	0.2546
	Rep(L)	153	1.289E-3	1.44	0.0018	0.130E-3	0.232E-3	0.232E-3	
	ϵ	537	0.896E-3	-	-	0.896E-3	1.014E-3	1.014E-3	
25 days old on restricted diet	L	86	3.105E-3	2.10	<0.0001	0.224E-3	0.088E-3	0.361E-3	0.1435
	Rep(L)	125	1.470E-3	1.10	0.2433	0.045E-3	-0.09E-3	0.182E-3	
	ϵ	443	1.337E-3	-	-	1.337E-3	1.177E-3	1.532E-3	
25 days old on both diets	D	1	0.3638	23.28	<0.0001	Fixed	Fixed	Fixed	0.1949
	L	96	4.744E-3	2.87	<0.0001	0.226E-3	0.120E-3	0.331E-3	
	D×L	84	1.654E-3	1.21	0.1329	0.039E-3	-0.04E-3	0.116E-3	
	Rep(A×D×L)	278	1.370E-3	1.25	0.0083	0.092E-3	8.871E-6	0.175E-3	
	ϵ	980	1.095E-3	-	-	1.095E-3	1.005E-3	1.199E-3	
7, 16 and 25 days old on both diets	A	2	6.195E-3	3.13	0.0460	Fixed	Fixed	Fixed	0.2594
	D	1	0.1611	72.88	<0.0001	Fixed	Fixed	Fixed	
	L	97	0.0136	5.87	<0.0001	0.267E-3	0.175E-3	0.359E-3	
	A×D	2	0.393E-3	0.20	0.8204	Fixed	Fixed	Fixed	
	A×L	191	2.050E-3	1.01	0.4820	0.989E-6	-0.04E-3	0.041E-3	
	D×L	97	2.289E-3	1.13	0.2408	0.013E-3	-0.02E-3	0.050E-3	
	A×D×L	176	2.039E-3	1.62	<0.0001	0.104E-3	0.044E-3	0.165E-3	
	Rep(A×D×L)	948	1.251E-3	1.14	0.0064	0.054E-3	0.011E-3	0.097E-3	
	ϵ	2990	1.099E-3	-	-	1.099E-3	1.046E-3	1.157E-3	

Table S5. Summary of full and reduced ANOVA models assessing locomtor activity of DGRP flies at 7, 16 and 25 days old and fed control and restricted diets. Diet and age were considered fixed effects, and DGRP line is a random effect.

Analysis	Source	DF	MS	F Value	*p*-value	σ^2	Lower	Upper	H^2
7 days old on control diet	L	97	3.7961	5.21	<0.0001	0.4034	0.2627	0.5442	0.3717
	Rep(L)	170	0.7225	1.06	0.3143	0.0152	-0.0505	0.0809	
	ϵ	502	0.6819	-	-	0.6819	0.6048	0.7748	
7 days old on restricted diet	L	97	5.3835	5.11	<0.0001	0.5805	0.3756	0.7855	0.4221
	Rep(L)	168	1.0182	1.28	0.0219	0.0848	-0.0055	0.1750	
	ϵ	492	0.7949	-	-	0.7949	0.7042	0.9044	
7 days old on both diets	D	1	0.0010	0.00	0.9807	Fixed	Fixed	Fixed	0.4004
	L	97	7.4081	4.20	<0.0001	0.3762	0.2328	0.5197	
	D×L	97	1.7623	1.98	<0.0001	0.1164	0.0488	0.1841	
	Rep(D×L)	338	0.8695	1.18	0.0299	0.0496	-0.0051	0.1043	
	ϵ	994	0.7378	-	-	0.7378	0.6770	0.8073	
	L	92	6.3577	5.14	<0.0001	0.8203	0.5191	1.1215	0.4533

16 days old on control diet	Rep(L)	144	1.2140	1.23	0.0642	0.0933	-0.0399	0.2265	
	ϵ	380	0.9892	-	-	0.9892	0.8624	1.1465	
16 days old on restricted diet	L	93	4.9050	5.84	<0.0001	0.6087	0.4005	0.8169	0.4006
	Rep(L)	161	0.8474	0.93	0.7010	-0.0263	-0.1091	0.0565	
	ϵ	417	0.9108	-	-	0.9108	0.7988	1.0483	
16 days old on both diets	D	1	34.0410	13.64	0.0004	Fixed	Fixed	Fixed	0.4315
	L	95	8.2341	3.09	<0.0001	0.4574	0.2560	0.6588	
	D×L	90	2.6653	2.60	<0.0001	0.2622	0.1345	0.3899	
	Rep(D×L)	305	1.0205	1.08	0.2150	0.0300	-0.0464	0.1063	
	ϵ	797	0.9482	-	-	0.9482	0.8616	1.0487	
7 and 16 days old on control diet	A	1	16.944	4.57	0.0351	Fixed	Fixed	Fixed	0.4281
	L	97	6.1006	1.53	0.0200	0.1616	0.0088	0.3144	
	A×L	92	3.9510	4.12	<0.0001	0.4479	0.2735	0.6222	
	Rep(A×L)	314	0.9479	1.16	0.0477	0.0524	-0.0156	0.1204	
	ϵ	882	0.8143	-	-	0.8143	0.7433	0.8960	
7 and 16 days old on restricted diet	A	1	1.1681	0.60	0.4417	Fixed	Fixed	Fixed	0.4126
	L	97	8.0454	3.98	<0.0001	0.4417	0.2691	0.6143	
	A×L	93	2.0150	2.14	<0.0001	0.1541	0.0714	0.2368	
	Rep(A×L)	329	0.9346	1.10	0.1380	0.0343	-0.0263	0.0948	
	ϵ	909	0.8481	-	-	0.8481	0.7752	0.9318	
7 and 16 days old on both diets	A	1	4.5364	1.38	0.2433	Fixed	Fixed	Fixed	0.4227
	D	1	18.8916	10.46	0.0016	Fixed	Fixed	Fixed	
	L	97	12.0457	4.42	<0.0001	0.3559	0.2177	0.4941	
	A×D	1	17.4111	7.01	0.0095	Fixed	Fixed	Fixed	
	A×L	95	3.4283	1.33	0.0848	0.0656	-0.0261	0.1573	
	D×L	97	1.8744	0.72	0.9413	-0.0553	-0.1224	0.0118	
	A×D×L	90	2.5778	2.71	<0.0001	0.2426	0.1282	0.3571	
	Rep(A×D×L)	643	0.9411	1.13	0.0266	0.0432	-0.0026	0.0890	
	ϵ	1791	0.8315	-	-	0.8315	0.7796	0.8887	
25 days old on control diet	L	91	5.8282	3.58	<0.0001	0.6632	0.3847	0.9418	0.4159
	Rep(L)	134	1.6315	1.75	<0.0001	0.2665	0.1154	0.4176	
	ϵ	392	0.9313	-	-	0.9313	0.8135	1.0767	
25 days old on restricted diet	L	86	2.8888	2.77	<0.0001	0.3037	0.1517	0.4556	0.2380
	Rep(L)	119	1.0438	1.07	0.3084	0.0275	-0.0808	0.1359	
	ϵ	351	0.9722	-	-	0.9722	0.8430	1.1337	
25 days old on both diets	D	1	0.9845	0.55	0.4583	Fixed	Fixed	Fixed	0.3446
	L	95	6.1768	3.19	<0.0001	0.3929	0.2112	0.5746	
	D×L	82	1.9615	1.47	0.0125	0.1070	-0.0091	0.2230	
	Rep(A×D×L)	253	1.3551	1.43	0.0002	0.1547	0.0629	0.2465	
	ϵ	743	0.9506	-	-	0.9506	0.8609	1.0552	
7, 16 and 25 days old on both diets	A	2	18.6262	6.22	0.0024	Fixed	Fixed	Fixed	0.3931
	D	1	14.7378	7.73	0.0064	Fixed	Fixed	Fixed	
	L	97	13.5681	4.33	<0.0001	0.3003	0.1906	0.4101	
	A×D	2	9.9269	4.91	0.0084	Fixed	Fixed	Fixed	
	A×L	190	3.2651	1.54	0.0020	0.0945	0.0279	0.1611	
	D×L	97	2.0550	0.97	0.5586	-0.0036	-0.0416	0.0345	
	A×D×L	172	2.1466	2.02	<0.0001	0.1700	0.0956	0.2444	
	Rep(A×D×L)	896	1.0580	1.22	0.0001	0.0749	0.0337	0.1160	
	ϵ	2534	0.8664	-	-	0.8664	0.8206	0.9162	

Table S6. Summary of full and reduced ANOVA models assessing HKDT of DGRP flies at 7, 16 and 25 days old and fed control and restricted diets. Diet and age were considered fixed effects, and DGRP line is a random effect.

Analysis	Source	DF	MS	F Value	*p*-value	σ^2	Lower	Upper	H^2
7 days old on control diet	L	96	531.8690	4.23	<0.0001	59.3788	36.9663	81.7913	0.3865
	Rep(L)	157	121.8875	1.29	0.0222	10.9662	-2.5497	24.4821	
	ϵ	437	94.2677	-	-	94.2677	82.9184	108.13	
7 days old on restricted diet	L	93	394.1051	3.60	<0.0001	51.3928	28.8399	73.9457	0.3264
	Rep(L)	138	109.0432	1.03	0.4159	1.3612	-13.0343	15.7566	
	ϵ	325	106.0603	-	-	106.06	91.4711	124.46	
7 days old on both diets	D	1	16006	58.94	<0.0001	Fixed	Fixed	Fixed	0.3613
	L	96	608.4325	2.06	0.0003	26.5633	9.7362	43.3904	
	D×L	93	292.9536	2.50	<0.0001	29.6048	15.4089	43.8007	

	Rep(D×L)	295	115.8790	1.17	0.0522	7.0095	-2.8255	16.8446	
	ϵ	762	99.2973	-	-	99.2973	90.0350	110.07	
16 days old on control diet	L	61	229.8621	1.35	0.1267	17.6157	-15.5556	50.7870	0.1137
	Rep(L)	57	170.3693	1.24	0.1695	18.2846	-36.9643	73.5335	
	ϵ	106	137.3836	-	-	137.38	106.78	183.41	
16 days old on restricted diet	L	83	250.4292	3.05	<0.0001	35.6428	16.2972	54.9883	0.3130
	Rep(L)	114	81.9705	1.05	0.3795	1.8777	-15.4180	19.1733	
	ϵ	232	78.2379	-	-	78.2379	65.7461	94.6823	
16 days old on both diets	D	1	1470.7298	7.26	0.0088	Fixed	Fixed	Fixed	0.2475
	L	86	230.6495	0.98	0.5364	-0.6597	-18.6607	17.3413	
	D×L	58	227.3544	2.05	0.0002	32.4862	4.2173	60.7551	
	Rep(D×L)	171	111.4368	1.15	0.1393	7.6041	-12.1045	27.3127	
	ϵ	338	96.7865	-	-	96.7865	83.7044	113.21	
7 and 16 days old on control diet	A	1	34808	181.80	<0.0001	Fixed	Fixed	Fixed	0.3512
	L	96	518.1841	2.21	0.0013	36.0583	10.3376	61.7790	
	A×L	61	212.7383	1.62	0.0054	19.5184	-0.6681	39.7049	
	Rep(A×L)	214	134.8009	1.31	0.0073	13.7940	-0.8023	28.3903	
	ϵ	543	102.6844	-	-	102.68	91.4866	116.08	
7 and 16 days old on restricted diet	A	1	6256.9640	35.56	<0.0001	Fixed	Fixed	Fixed	0.3293
	L	94	440.3188	2.33	<0.0001	27.4449	10.2982	44.5916	
	A×L	82	189.9159	1.96	<0.0001	18.9446	6.3002	31.5890	
	Rep(A×L)	252	96.7960	1.02	0.4051	1.1071	-9.4287	11.6430	
	ϵ	557	94.4718	-	-	94.4718	84.2890	106.63	
7 and 16 days old on both diets	A	1	34579	207.86	<0.0001	Fixed	Fixed	Fixed	0.3234
	D	1	1130.5781	4.83	0.0298	Fixed	Fixed	Fixed	
	L	96	558.1189	2.18	0.0157	19.4587	6.4627	32.4547	
	A×D	1	7162.0576	36.57	<0.0001	Fixed	Fixed	Fixed	
	A×L	86	182.3947	0.87	0.7284	-3.8503	-14.3266	6.6260	
	D×L	94	286.7512	1.31	0.1426	9.1820	-4.7773	23.1412	
	A×D×L	57	210.4926	1.85	0.0003	22.3075	5.4590	39.1559	
	Rep(A×D×L)	466	114.2489	1.16	0.0273	7.1323	-1.6360	15.9005	
	ϵ	1100	98.5258	-	-	98.5258	90.7837	107.31	
25 days old on control diet	L	69	279.3379	2.47	<0.0001	44.1435	16.9072	71.3799	0.2263
	Rep(L)	67	110.9311	151	0.9224	-19.8269	-40.4556	0.8018	
	ϵ	151	150.9137	-	-	150.91	121.92	191.70	
25 days old on restricted diet	L	73	254.5007	1.44	0.0577	19.0153	-1.6391	39.6697	0.2059
	Rep(L)	79	177.7359	2.42	<0.0001	52.3019	20.3548	84.2490	
	ϵ	167	73.3219	-	-	73.3219	59.8254	91.9893	
25 days old on both diets	D	1	414.8376	1.77	0.1874	Fixed	Fixed	Fixed	0.2257
	L	84	259.2257	1.00	0.5112	-0.1370	-20.7342	20.4603	
	D×L	58	262.7501	1.81	0.0020	32.2515	6.8154	57.6875	
	Rep(A×D×L)	146	147.0789	1.34	0.0182	18.4046	-2.0600	38.8692	
	ϵ	318	110.1658	-	-	110.17	94.8639	129.51	
7, 16 and 25 days old on both diets	A	2	25938	140.32	<0.0001	Fixed	Fixed	Fixed	0.2974
	D	1	189.4276	0.75	0.3870	Fixed	Fixed	Fixed	
	L	96	546.6994	1.74	0.0143	11.9537	1.5929	22.3146	
	A×D	2	5102.5452	26.87	<0.0001	Fixed	Fixed	Fixed	
	A×L	170	206.2055	1.02	0.4686	0.4400	-8.4650	9.3450	
	D×L	94	309.9370	1.51	0.0165	11.3762	1.5052	21.2471	
	A×D×L	115	205.9576	1.70	<0.0001	20.0453	7.3953	32.6954	
	Rep(A×D×L)	612	122.0809	1.21	0.0026	9.7098	1.4948	17.9248	
	ϵ	1418	101.1362	-	-	101.14	94.0859	109.01	

Table S7. Summary of full and reduced ANOVA models for balanced data assessing dry weight of DGRP flies at 7, 16 and 25 days old and fed control and restricted diets. Diet and age were considered fixed effects, and DGRP line is a random effect.

Analysis	Source	DF	MS	F Value	*p*-value	σ^2	Lower	Upper	H^2
7 days old on control diet	L	97	6.578E-3	5.03	<0.0001	0.686E-3	0.442E-3	0.929E-3	0.3437
	Rep(L)	170	1.308E-3	1.00	0.4956	-0.594E-6	-0.11E-3	0.106E-3	
	ϵ	510	1.310E-3	-	-	1.310E-3	1.163E-3	1.487E-3	
7 days old on restricted diet	L	97	3.885E-3	2.96	<0.0001	0.332E-3	0.185E-3	0.478E-3	0.2542
	Rep(L)	169	1.267E-3	1.30	0.0155	0.107E-3	-3.03E-6	0.217E-3	
	ϵ	516	0.974E-3	-	-	0.974E-3	0.866E-3	1.105E-3	

7 days old on both diets	D	1	0.0761	26.61	<0.0001	Fixed	Fixed	Fixed	0.3081
	L	97	7.572E-3	2.63	<0.0001	0.304E-3	0.156E-3	0.452E-3	
	D×L	97	2.88E-3	2.20	<0.0001	0.204E-3	0.092E-3	0.315E-3	
	Rep(D×L)	339	1.288	1.13	0.0823	0.054E-3	-0.02E-3	0.131E-3	
	ϵ	1026	1.141E-3	-	-	1.141E-3	1.049E-3	1.247E-3	
16 days old on control diet	L	92	3.580E-3	3.35	<0.0001	0.325E-3	0.187E-3	0.464E-3	0.2395
	Rep(L)	164	1.066E-3	1.03	0.3926	0.012E-3	-0.08E-3	0.102E-3	
	ϵ	483	1.032E-3	-	-	1.032E-3	0.914E-3	1.176E-3	
16 days old on restricted diet	L	92	4.369E-3	3.69	<0.0001	0.419E-3	0.250E-3	0.588E-3	0.2779
	Rep(L)	162	1.173E-3	1.08	0.2741	0.031E-3	-0.07E-3	0.132E-3	
	ϵ	485	1.089E-3	-	-	1.089E-3	0.964E-3	1.241E-3	
16 days old on both diets	D	1	0.0560	31.80	<0.0001	Fixed	Fixed	Fixed	0.2580
	L	92	6.126E-3	3.46	<0.0001	0.285E-3	0.164E-3	0.406E-3	
	D×L	92	1.770E-3	1.57	0.0030	0.084E-3	0.014E-3	0.154E-3	
	Rep(D×L)	326	1.119E-3	1.05	0.2718	0.022E-3	-0.04E-3	0.088E-3	
	ϵ	968	1.061E-3	-	-	1.061E-3	0.972E-3	1.162E-3	
7 and 16 days old on control diet	A	1	1.080E-3	0.50	0.4801	Fixed	Fixed	Fixed	0.3095
	L	92	8.166E-3	3.80	<0.0001	0.391E-3	0.230E-3	0.552E-3	
	A×L	92	2.151E-3	1.86	<0.0001	0.129E-3	0.045E-3	0.214E-3	
	Rep(A×L)	329	1.156E-3	1.00	0.5109	-1.63E-6	-0.07E-3	0.066E-3	
	ϵ	964	1.160E-3	-	-	1.160E-3	1.063E-3	1.271E-3	
7 and 16 days old on restricted diet	A	1	2.790E-3	1.28	0.2610	Fixed	Fixed	Fixed	0.2588
	L	92	5.845E-3	2.65	<0.0001	0.238E-3	0.120E-3	0.356E-3	
	A×L	92	2.203E-3	1.75	0.0003	0.123E-3	0.040E-3	0.207E-3	
	Rep(A×L)	326	1.234E-3	1.19	0.0233	0.074E-3	-3.67E-6	0.151E-3	
	ϵ	971	1.034E-3	-	-	1.034E-3	0.948E-3	1.133E-3	
7 and 16 days old on both diets	A	1	3.387E-3	1.62	0.2062	Fixed	Fixed	Fixed	0.2871
	D	1	0.1252	52.12	<0.0001	Fixed	Fixed	Fixed	
	L	92	0.0116	5.18	<0.0001	0.304E-3	0.188E-3	0.420E-3	
	A×D	1	0.191E-3	0.08	0.7723	Fixed	Fixed	Fixed	
	A×L	92	2.096E-3	0.92	0.6517	-0.01E-3	-0.07E-3	0.046E-3	
	D×L	92	2.410E-3	1.06	0.3912	8.824E-6	-0.05E-3	0.071E-3	
	A×D×L	92	2.275E-3	1.89	<0.0001	0.139E-3	0.051E-3	0.228E-3	
	Rep(A×D×L)	655	1.195E-3	1.09	0.0881	0.036E-3	-0.02E-3	0.087E-3	
	ϵ	1935	1.097E-3	-	-	1.097E-3	1.031E-3	1.170E-3	
25 days old on control diet	L	84	3.944E-3	2.92	<0.0001	0.316E-3	0.163E-3	0.469E-3	0.2631
	Rep(L)	143	1.324E-3	1.50	0.0009	0.146E-3	0.037E-3	0.254E-3	
	ϵ	490	0.885E-3	-	-	0.885E-3	0.783E-3	1.007E-3	
25 days old on restricted diet	L	84	3.177E-3	2.14	<0.0001	0.232E-3	0.091E-3	0.373E-3	0.1476
	Rep(L)	124	1.482E-3	1.11	0.2319	0.048E-3	-0.09E-3	0.186E-3	
	ϵ	433	1.340E-3	-	-	1.340E-3	1.178E-3	1.538E-3	
25 days old on both diets	D	1	0.0364	23.27	<0.0001	Fixed	Fixed	Fixed	0.1985
	L	84	5.166E-3	3.12	<0.0001	0.237E-3	0.125E-3	0.350E-3	
	D×L	84	1.654E-3	1.18	0.1608	0.035E-3	-0.04E-3	0.113E-3	
	Rep(A×D×L)	267	1.397E-3	1.27	0.0058	0.100E-3	0.014E-3	0.186E-3	
	ϵ	923	1.098E-3	-	-	1.098E-3	1.005E-3	1.206E-3	
7, 16 and 25 days old on both diets	A	2	1.036E-3	0.81	0.4453	Fixed	Fixed	Fixed	0.9763
	D	1	0.063E-9	0.00	0.9998	Fixed	Fixed	Fixed	
	L	82	0.0136	-0.01	-	0.0247	0.0234	0.0260	
	A×D	2	0.526E-3	0.00	0.9991	Fixed	Fixed	Fixed	
	A×L	164	2.111E-3	0.00	1.0000	-0.0734	-0.0772	-0.0696	
	D×L	82	2.400E-3	0.00	1.0000	-0.0489	-0.0514	-0.0464	
	A×D×L	164	2.0827	1649.96	<0.0001	0.1467	0.1391	0.1543	
	Rep(A×D×L)	859	1.252E-3	1.12	0.0208	0.047E-3	4.1E-6	0.091E-3	
	ϵ	2636	1.119E-3	-	-	1.119E-3	1.061E-3	1.182E-3	

Table S8. Summary of full and reduced ANOVA models for balanced data assessing locomotor activity of DGRP flies at 7, 16 and 25 days old and fed control and restricted diets. Diet and age were considered fixed effects, and DGRP line is a random effect.

Analysis	Source	DF	MS	F Value	*p*-value	σ^2	Lower	Upper	H^2
7 days old on control diet	L	97	3.7961	5.21	<0.0001	0.4034	0.2627	0.5442	0.3717
	Rep(L)	170	0.7225	1.06	0.3143	0.0152	-0.0505	0.0809	

	ϵ	502	0.6819	-	-	0.6819	0.6048	0.7748	
7 days old on restricted diet	L	97	5.3835	5.11	<0.0001	0.5805	0.3756	0.7855	0.4221
	Rep(L)	168	1.0182	1.28	0.0219	0.0848	-0.0055	0.1750	
	ϵ	492	0.7949	-	-	0.7949	0.7042	0.9044	
7 days old on both diets	D	1	0.0010	0.00	0.9807	Fixed	Fixed	Fixed	0.4004
	L	97	7.4081	4.20	<0.0001	0.3762	0.2328	0.5197	
	D×L	97	1.7623	1.98	<0.0001	0.1164	0.0488	0.1841	
	Rep(D×L)	338	0.8695	1.18	0.0299	0.0496	-0.0051	0.1043	
	ϵ	994	0.7378	-	-	0.7378	0.6770	0.8073	
16 days old on control diet	L	90	5.8590	4.64	<0.0001	0.7390	0.4590	1.0190	0.4250
	Rep(L)	141	1.2394	1.24	0.0575	0.0998	-0.0366	0.2361	
	ϵ	370	1.0000	-	-	1.0000	0.8702	1.1614	
16 days old on restricted diet	L	90	4.8761	5.74	<0.0001	0.5939	0.3863	0.8014	0.3942
	Rep(L)	156	0.8563	0.94	0.6761	-0.0231	-0.1077	0.0616	
	ϵ	413	0.9127	-	-	0.9127	0.8000	1.0512	
16 days old on both diets	D	1	34.0410	13.64	0.0004	Fixed	Fixed	Fixed	0.4126
	L	90	7.8453	2.93	<0.0001	0.4108	0.2248	0.5969	
	D×L	90	2.6653	2.55	<0.0001	0.2593	0.1307	0.3878	
	Rep(D×L)	297	1.0382	1.09	0.1848	0.0348	-0.0435	0.1130	
	ϵ	783	0.9540	-	-	0.9540	0.8661	1.0560	
7 and 16 days old on control diet	A	1	18.4781	5.14	0.0258	Fixed	Fixed	Fixed	0.4179
	L	90	6.0150	1.56	0.0186	0.1604	0.0066	0.3141	
	A×L	90	3.8375	3.90	<0.0001	0.4286	0.2576	0.5996	
	Rep(A×L)	302	0.9706	1.18	0.0354	0.0590	-0.0123	0.1303	
	ϵ	836	0.8204	-	-	0.8204	0.7471	0.9051	
7 and 16 days old on restricted diet	A	1	2.0177	1.01	0.3168	Fixed	Fixed	Fixed	0.4073
	L	90	8.0030	3.91	<0.0001	0.4236	0.2510	0.5961	
	A×L	90	2.0448	2.23	<0.0001	0.1603	0.0766	0.2440	
	Rep(A×L)	315	0.9114	1.07	0.2198	0.0243	-0.0357	0.0842	
	ϵ	870	0.8496	-	-	0.8496	0.7751	0.9354	
7 and 16 days old on both diets	A	1	4.1015	1.26	0.2641	Fixed	Fixed	Fixed	0.4140
	D	1	21.2443	11.45	0.0010	Fixed	Fixed	Fixed	
	L	90	11.8439	4.38	<0.0001	0.3387	0.2019	0.4755	
	A×D	1	17.4111	7.00	0.0096	Fixed	Fixed	Fixed	
	A×L	90	3.3723	1.31	0.1044	0.0586	-0.0314	0.1486	
	D×L	90	1.9098	0.74	0.9235	-0.0501	-0.1200	0.0197	
	A×D×L	90	2.5778	2.72	<0.0001	0.2429	0.1271	0.3586	
	Rep(A×D×L)	617	0.9404	1.13	0.0352	0.0413	-0.0056	0.0880	
	ϵ	1706	0.8353	-	-	0.8353	0.7820	0.8943	
25 days old on control diet	L	82	5.9119	3.76	<0.0001	0.6601	0.3753	0.9448	0.4154
	Rep(L)	126	1.5622	1.68	<0.0001	0.2395	0.0919	0.3872	
	ϵ	370	0.9290	-	-	0.9290	0.8084	1.0788	
25 days old on restricted diet	L	82	2.9789	2.78	<0.0001	0.3083	0.1512	0.4654	0.2404
	Rep(L)	115	1.0713	1.10	0.2566	0.0372	-0.0761	0.1504	
	ϵ	343	0.9741	-	-	0.9741	0.8433	1.1380	
25 days old on both diets	D	1	0.9845	0.55	0.4582	Fixed	Fixed	Fixed	0.3436
	L	82	6.5560	3.32	<0.0001	0.3859	0.2017	0.5701	
	D×L	82	1.9615	1.50	0.0093	0.1118	-0.0045	0.2281	
	Rep(A×D×L)	241	1.3279	1.40	0.0005	0.1434	0.0519	0.2350	
	ϵ	713	0.9507	-	-	0.9507	0.8592	1.0576	
7, 16 and 25 days old on both diets	A	2	18.2214	5.81	0.0037	Fixed	Fixed	Fixed	0.3782
	D	1	16.7769	7.87	0.0062	Fixed	Fixed	Fixed	
	L	79	14.3821	4.28	<0.0001	0.2863	0.1684	0.4041	
	A×D	2	5.3980	2.64	0.0747	Fixed	Fixed	Fixed	
	A×L	158	3.3132	1.54	0.0037	0.0881	0.0208	0.1553	
	D×L	79	2.2244	1.05	0.3943	0.0054	-0.0380	0.0488	
	A×D×L	158	2.1464	2.00	<0.0001	0.1647	0.0886	0.2408	
	Rep(A×D×L)	796	1.0652	1.19	0.0012	0.0661	0.0221	0.1100	
	ϵ	2228	0.8950	-	-	0.8950	0.8447	0.9500	

Table S9. Summary of full and reduced ANOVA models for balanced data assessing HKDT of DGRP flies at 7, 16 and 25 days old and fed control and restricted diets. Diet and age were considered fixed effects, and DGRP line is a random effect.

Analysis	Source	DF	MS	F Value	*p*-value	σ^2	Lower	Upper	H^2

7 days old on control diet	L	93	490.7827	3.85	<0.0001	53.1426	31.8696	74.4157	0.3636
	Rep(L)	151	123.3621	1.33	0.0150	12.0271	-1.7060	25.7602	
	ϵ	424	93.0129	-	-	93.0129	81.6604	106.92	
7 days old on restricted diet	L	93	394.1051	3.60	<0.0001	51.3928	28.8399	73.9457	0.3264
	Rep(L)	138	109.0432	1.03	0.4159	1.3612	-13.0343	15.7566	
	ϵ	325	106.0603	-	-	106.06	91.4711	124.46	
7 days old on both diets	D	1	16006	58.96	<0.0001	Fixed	Fixed	Fixed	0.3475
	L	93	569.9046	1.94	0.0008	23.0790	7.6422	38.5158	
	D×L	93	292.9536	2.48	<0.0001	29.4762	15.1484	43.8040	
	Rep(D×L)	289	116.5247	1.18	0.0417	7.5481	-2.3807	17.4769	
	ϵ	749	98.6743	-	-	98.6743	89.3964	109.48	
16 days old on control diet	L	58	231.8209	1.38	0.1179	18.8209	-14.1136	51.7554	0.1188
	Rep(L)	55	168.2373	1.21	0.2082	16.0936	-35.9378	68.1249	
	ϵ	100	139.6117	-	-	139.61	107.76	188.10	
16 days old on restricted diet	L	58	267.0131	3.02	<0.0001	35.4535	12.4704	58.4367	0.3224
	Rep(L)	83	86.7528	1.16	0.2004	6.0470	-14.2512	26.3451	
	ϵ	180	74.4996	-	-	74.4996	61.2202	92.6476	
16 days old on both diets	D	1	1470.7298	7.25	0.0088	Fixed	Fixed	Fixed	0.2484
	L	58	247.4582	1.06	0.4117	1.9382	-17.3029	21.1793	
	D×L	58	227.3544	1.92	0.0010	30.3665	0.6431	60.0899	
	Rep(D×L)	138	119.2285	1.22	0.0839	11.1404	-11.4137	33.6944	
	ϵ	280	97.7539	-	-	97.7539	83.3861	116.21	
7 and 16 days old on control diet	A	1	32572	162.97	<0.0001	Fixed	Fixed	Fixed	0.2540
	L	57	341.5793	1.49	0.0724	12.8513	-5.1703	30.8729	
	A×L	57	222.6583	1.62	0.0091	20.7474	-2.3855	43.8802	
	Rep(A×L)	152	140.9800	1.43	0.0034	18.6465	-0.0421	37.3351	
	ϵ	377	98.6855	-	-	98.6855	85.9845	114.44	
7 and 16 days old on restricted diet	A	1	6631.1441	33.53	<0.0001	Fixed	Fixed	Fixed	0.3503
	L	57	431.9360	2.00	0.0050	20.4265	2.5801	38.2729	
	A×L	57	214.0833	2.39	<0.0001	23.7360	7.0134	40.4587	
	Rep(A×L)	172	89.0259	1.09	0.2514	3.3026	-8.5758	15.1810	
	ϵ	407	81.8909	-	-	81.8909	71.7092	94.4185	
7 and 16 days old on both diets	A	1	36098	187.00	<0.0001	Fixed	Fixed	Fixed	0.3000
	D	1	253.0245	0.95	0.3323	Fixed	Fixed	Fixed	
	L	57	426.9033	1.45	0.1454	7.3664	-5.5699	20.3027	
	A×D	1	7162.0576	36.83	<0.0001	Fixed	Fixed	Fixed	
	A×L	57	209.3845	0.98	0.5233	-0.3679	-14.0096	13.2737	
	D×L	57	294.8821	1.38	0.1186	8.9778	-6.7782	24.7339	
	A×D×L	57	210.4926	1.87	0.0004	22.5833	3.7607	41.4059	
	Rep(A×D×L)	324	113.3995	1.26	0.0057	10.5982	-0.0089	21.2054	
	ϵ	784	89.9669	-	-	89.9669	81.6851	99.5820	
25 days old on control diet	L	58	266.8686	2.47	0.0002	39.8890	14.5473	65.2308	0.2098
	Rep(L)	61	107.0241	0.71	0.9318	-21.3864	-40.1835	-2.5894	
	ϵ	137	150.2157	-	-	150.22	120.14	193.25	
25 days old on restricted diet	L	58	218.8378	1.06	0.4060	3.0713	-16.0853	22.2279	0.0430
	Rep(L)	65	201.4163	2.94	<0.0001	67.1812	27.8347	106.53	
	ϵ	137	68.4057	-	-	68.4057	54.7105	88.0039	
25 days old on both diets	D	1	414.8376	1.77	0.1874	Fixed	Fixed	Fixed	0.1737
	L	58	214.3308	0.80	0.7957	-7.0070	-23.1949	9.1808	
	D×L	58	262.7501	1.71	0.0058	29.9785	3.7040	56.2530	
	Rep(A×D×L)	126	155.7185	1.42	0.0085	23.2143	-0.1832	46.6118	
	ϵ	274	109.3107	-	-	109.31	93.0895	130.20	
7, 16 and 25 days old on both diets	A	2	21254	111.19	<0.0001	Fixed	Fixed	Fixed	0.2711
	D	1	27.2007	0.08	0.7829	Fixed	Fixed	Fixed	
	L	43	395.7983	1.04	0.4640	0.5485	-9.9679	11.0649	
	A×D	2	3430.1651	16.71	<0.0001	Fixed	Fixed	Fixed	
	A×L	86	206.4383	0.93	0.6400	-1.8587	-13.0767	9.3592	
	D×L	43	395.9592	1.82	0.0092	13.8909	-0.7253	28.5071	
	A×D×L	86	220.9922	1.85	<0.0001	22.9778	5.8859	40.0697	
	Rep(A×D×L)	359	120.7862	1.26	0.0038	11.4927	1.3147	21.6706	
	ϵ	838	95.5975	-	-	95.5975	87.0658	105.45	

Table S11. Summary of full and reduced ANOVA models assessing lifespan of DGRP flies fed control and restricted diets. Diet and age were considered fixed effects, and DGRP line is a random effect.

Analysis	Source	DF	MS	F Value	*p*-value	σ^2	Lower	Upper	H^2
Control diet	L	95	7601.7568	13.39	<0.0001	43.6137	29.4308	57.7966	0.3909
	Rep(L)	171	600.3582	8.83	<0.0001	8.8832	6.8248	10.9416	
	ϵ	15637	67.9693	-	-	67.9693	66.4875	69.5014	
Restricted diet	L	97	7401.8553	14.06	<0.0001	35.5377	24.5383	46.5371	0.5319
	Rep(L)	171	565.1848	18.07	<0.0001	7.3473	5.6598	9.0349	
	ϵ	18894	31.2792	-	-	31.2792	30.6579	31.9197	
Both diets	D	1	21019	8.65	0.0041	Fixed	Fixed	Fixed	0.4525
	L	97	11589	3.79	<0.0001	24.8539	14.5188	35.1891	
	D×L	95	3120.5366	5.77	<0.0001	14.7233	9.4495	19.9971	
	Rep(D×L)	342	582.7715	12.17	<0.0001	8.0676	6.7788	9.3565	
	ϵ	34531	47.8939	-	-	47.8939	47.1875	48.6164	
